# Integrated metabolomics and transcriptomics investigate the bulb-specific biosynthesis of medicinal steroidal alkaloids in *Fritillaria taipaiensis*

**DOI:** 10.1186/s12870-026-08432-x

**Published:** 2026-02-28

**Authors:** Jiaqi Guo, Xingchi Zhong, Le Zhou, Keke Chen, Mian Wei, Xinyu Gao, Shijie Wang, Zengqiang Qian, Langjun Cui, Yi Qiang, Hongyan Ren

**Affiliations:** 1 National Engineering Laboratory for Resource Development of Endangered Chinese Crude Drugs in Northwest of China, Shaanxi Normal University, Xi’an, 710119 China; 2 Key Laboratory of Medicinal Resources and Natural Pharmaceutical Chemistry, The Ministry of Education, Shaanxi Normal University, Xi’an, 710119 China; 3 College of Life Sciences, Shaanxi Normal University, Xi’an, 710119 China; 4 School of Biological and Environmental Engineering, Xi’an University, Xi’an, 710065 China

**Keywords:** Fritillaria taipaiensis, Steroidal alkaloids, Enzyme subfunctionalization, Metabolic engineering, Breeding

## Abstract

**Background:**

*Fritillaria taipaiensis* is a commercially important medicinal herb, the bulbs of which are a valued source of steroidal alkaloids with potent antitussive and expectorant properties. However, the mechanisms underlying the tissue-specific biosynthesis of these bioactive compounds remain poorly understood, limiting strategies for their enhanced production.

**Results:**

This study, we integrated UPLC-MS/MS-based metabolomics with multi-tissue transcriptomics to elucidate the steroidal alkaloid biosynthetic pathway in *F. taipaiensis*. Comparative metabolomic profiling revealed distinct tissue-specific accumulation patterns, with bulbs serving as the primary site for therapeutically relevant steroidal alkaloids such as peimisine, edpetiline, and delafrine. Transcriptomic analysis unveiled metabolic compartmentalization, characterized by the bulb-specific upregulation of mevalonate (MVA) pathway genes, contrasting with leaf-predominant expression of methylerythritol phosphate (MEP) pathway components. This indicates a tissue-specific partitioning of precursor supply. Of note, we identified bulb-specific isoforms *FrtSSR1* (encoding a Δ²⁴(²⁵)-reductase) and *FrtSMO1-1* (encoding a C-4 demethylase), which were highly expressed and are proposed to pivotal roles in redirecting metabolic flux toward cholesterol biosynthesis. Furthermore, the coordinated induction of cytochrome P450 enzymes in bulbs suggests their involvement in mediating structural diversification of the steroidal skeleton.

**Conclusions:**

Our findings systematically unravel the molecular basis of bulb-specific steroidal alkaloid accumulation, highlighting pathway specialization, enzyme subfunctionalization, and CYP450-mediated modification. This study provides a genetic foundation and key candidate genes for future metabolic engineering and molecular breeding strategies aimed at improving the yield of high-value steroidal alkaloids in *Fritillaria* species.

**Supplementary Information:**

The online version contains supplementary material available at 10.1186/s12870-026-08432-x.

## Introduction

For over two millennia, the bulbs from *Fritillaria* species (Liliaceae), particularly *Fritillaria cirrhosa* D. Don, have been integral to traditional Chinese medicine for treating respiratory ailments, pulmonary fibrosis, and throat infections [1, 2]. Their medicinal properties are largely attributed to steroidal alkaloids such as peimine, peiminine, and sipeimine, which demonstrate antitussive, expectorant, and anti-inflammatory activities [3–5]. *F. taipaiensis* P.Y. Li serve as a critical genetic resource for *F. cirrhosa* and has been traditionally used to address lung-heat syndrome and dry cough [1, 6, 7]. This species exhibits broad ecological adaptability, thriving in alpine environments at elevations between 1800 and 3150 m [8].

The growing commercial demand for *Fritillaria* bulbs, driven by their extensive use in traditional medicine and pharmaceutical industries, has led to overharvesting and severe depletion of wild populations [8, 9]. Conventional cultivation methods are often slow and insufficient to meet market needs, highlighting an urgent necessity for developing innovative biotechnological strategies, such as metabolic engineering or in vitro biosynthesis, for sustainable and efficient production of these high-value compounds [10, 11]. Nevertheless, progress in this area remains constrained by an incomplete understanding of the steroidal alkaloid biosynthetic pathways and their regulation [12, 13].

Structurally, steroidal alkaloids are characterized by a cyclopentanoperhydrophenanthrene core modified by the incorporation of a basic nitrogen atom into heterocyclic rings or alkylamine side chains [13, 14]. Their biosynthesis begins with isoprenoid precursors from the mevalonate (MVA) and methylerythritol phosphate (MEP) pathways, which converge at farnesyl diphosphate (FPP) [13, 15]. FPP is subsequently converted to squalene and cyclized by cycloartenol synthase (CAS) to form cycloartenol—a key precursor for both cholesterol and C-24 alkyl phytosterols [16, 17]. Cholesterol serves as the central precursor for diverse specialized metabolites, including steroidal alkaloids and glycoalkaloids [18–20], with metabolic flux through cholesterol biosynthesis directly influencing alkaloid production [21, 22].

Cholesterol biosynthesis in plants involves approximately 12 enzymes catalyzing 10 steps, including both lineage-specific enzymes and multifunctional enzymes shared with phytosterol biosynthesis. It is widely accepted that several cholesterol-specific enzymes evolved from phytosterol biosynthetic enzymes via gene duplication and functional divergence [21]. Key regulatory nodes include sterol side chain reductases (SSRs)—where SSR1 functions primarily in phytosterol synthesis and SSR2 directs metabolic flux toward cholesterol [23]—and distinct sterol C-4 methyloxidases (SMOs): SMO1/SMO2 for phytosterol biosynthesis and SMO3/SMO4 for cholesterol formation [21]. Silencing SMO3 and SMO4 in tomato significantly reduced cholesterol and steroidal alkaloid levels while causing intermediates to accumulate [21]. Other enzymes, including CYP51, C14-sterol reductase (C14-R), and Δ8-Δ7-sterol isomerase (8,7-SI), function in both cholesterol and phytosterol pathways [21, 24].

The structural diversification of steroidal alkaloids occurs through post-cholesterol modifications, although these mechanisms remain poorly understood [25–27]. Cytochrome P450 monooxygenases (CYP450s) play a pivotal role in generating structural diversity through various reactions including hydroxylation, epoxidation, and carbon-carbon bond cleavage [28, 29]. The CYP71 family, for example, contributes to the diversification of monoterpenoid indole alkaloids through hydroxylation and epoxidation, as seen with tabersonine oxidation by CYP71D12 and CYP71D1 in *Catharanthus roseus* [30]. The CYP72 family catalyzes key ring-opening steps; CYP72A1 converts loganin to secologanin in *C. roseus* [31], while CYP72A565 and CYP72A610 in *Camptotheca acuminata* perform hydroxylation and subsequent ring cleavage to form secologanic acid [32]. Other CYP450s demonstrate remarkable functional diversity: CYP96T1 from *Narcissus* sp. aff. *pseudonarcissus* catalyzes phenolic coupling of 4′-O-methylnorbelladine [33], and CYP76AH subfamily members in *Isodon lophanthoides* mediate site-specific hydroxylations in diterpenoid biosynthesis [34]. Despite their established roles in alkaloid diversification across species, the specific CYP450 isoforms involved in steroidal alkaloid biosynthesis in *Fritillaria* remain largely uncharacterized.

A major unresolved question concerns the tissue-specific accumulation of steroidal alkaloids and the regulatory mechanisms controlling their biosynthesis. To address this knowledge gap and identify key genetic components for future pathway engineering, we combined UPLC-MS/MS-based metabolomics and full-length transcriptome sequencing to analyze *F. taipaiensis* bulbs and leaves. This integrated multi-omics approach is powerful for candidate gene identification and metabolic mapping in non-model species [35, 36]. Our specific objectives were to: (1) characterize the tissue-specific distribution and composition of alkaloids in *F. taipaiensis*; (2) identify bulb-enriched genes involved in steroidal alkaloid biosynthesis, with emphasis on the MVA/MEP pathways, SSRs, SMOs, and CYP450s; and (3) investigate the evolutionary divergence and functional specialization of these enzymes via phylogenetic analysis. This study not only provides critical insights into the metabolic basis of steroidal alkaloid accumulation but also offers a genetic toolkit and foundational knowledge for the quality improvement and biotechnological production of Fritillaria-based medicinal compounds.

## Materials and methods

### Plant materials

Bulbs and leaves of *F. taipaiensis* were collected in May 2023 from Taibai County, Baoji City, Shaanxi Province (107.38281°E, 34.04°N, altitude 1607.52 m). The plant materials were formally identified by Dr. Yi Qiang of Shaanxi Normal University. A voucher specimen (Specimen Number: GUO20230516001) has been deposited at the Herbarium of Shaanxi Normal University (SANU) for public access. The plant tissues were stored at -80 °C following collection. A total of six independent biological replicates were collected, with each replicate representing an entire individual plant. Bulb and leaf tissues were sampled from each replicate. For transcriptome library construction, RNA was extracted separately from the bulb and leaf tissues of three biological replicates. For metabolomic analysis, six replicates were utilized.Additionally, to acquire a comprehensive full-length transcriptome that covers the transcriptional information of different plant organs, roots, stems, leaves, flowers, and bulbs were collected from a single representative plant individual for full-length transcriptome sequencing. *Nicotiana benthamiana* plants were grown in a growth chamber with the temperature maintained at 23 ± 2 °C, under light intensity meeting the experimental requirements, and a photoperiod of 16 h light/8 h dark.

### Metabolite extraction

Biological samples were freeze-dried using a vacuum lyophilizer (Scientz-100 F) and subsequently ground to powder by using a grinder (MM 400, Retsch) at 30 Hz for 1.5 min. Exactly 50 mg of the powdered sample was weighed using an analytical balance (MS105DΜ) and extracted with 1200 µL of -20 °C pre-cooled 70% methanolic aqueous internal standard extract. The mixture was vortexed for 30 s at 30-minute intervals, for a total of 6 times. After centrifugation at 12,000 rpm for 3 min, the sample was filtered through a 0.22 μm microporous membrane and stored in the injection vial for UPLC-MS/MS analysis.

### UPLC conditions

The sample extracts were analyzed using an UPLC-ESI-MS/MS system (UPLC, ExionLC™ ADˈ https://sciex.com.cn/) and Tandem mass spectrometry (http://sciex.com.cn/). Separation was performed on an Agilent SB-C18 column (100 mm × 2.1 mm, 1.8 μm) maintained at 40 °C. The mobile phase consisted of solvent A (0.1% formic acid in water) and solvent B (0.1% formic acid in acetonitrile), with a gradient elution program as follows: 95% A and 5% B initially, ramped linearly to 5% A and 95% B over 9 min, held for 1 min, then returned to 95% A and 5% B in 1.1 min and held for 2.9 min for re-equilibration. The flow rate was 0.35 mL/min and the injection volume was 2 µL. The column effluent was directed into an ESI-triple quadrupole-linear ion trap (QTRAP) mass spectrometer for detection.

### ESI-Q TRAP-MS/MS

The operational parameters for the electrospray ionization (ESI) source were set as follows: source temperature, 500 °C; ion spray voltage (IS), 5500 V in positive ion mode and − 4500 V in negative ion mode; ion source gas I (GSI), 50 psi; ion source gas II (GSII), 60 psi; curtain gas (CUR), 25 psi; and collision-activated dissociation (CAD), high. Multiple reaction monitoring (MRM) scans were performed using nitrogen as the collision gas at medium pressure. Declustering potential (DP) and collision energy (CE) were individually optimized for each MRM transition. To ensure specific detection, a dedicated set of MRM transitions was monitored in each time segment based on the elution profile of the target metabolites.

### Metabolite qualitative annotation and quantification

Metabolite profiling was performed using an integrated approach combining untargeted and targeted metabolomics strategies. Qualitative analysis of mixed samples was carried out on an AB Sciex TripleTOF 6600 mass spectrometer, while quantitative analysis was conducted using an AB Sciex 4500 QTRAP system [37]. Metabolite identification were based on the MetWare database (MWDB, MetWare Biotechnology Co., Ltd., Wuhan, China). MWDB was constructed using authentic standards analyzed under consistent chromatographic and mass spectrometric conditions to obtain reference MS/MS spectra and RT values. For metabolites without commercial standards, public databases and literature were referenced, and in some cases, fragmentation patterns were inferred based on structural analogies and empirical rules.

To ensure high-confidence annotation, metabolite identification was performed by matching multiple parameters, including precursor mass (mass error ≤ 20 ppm), MS/MS spectral fragments, retention time (RT error ≤ 0.2 min), and isotopic distribution. The confidence of identification was classified into three levels according to the following criteria: Level 1: Confident identification, based on a high spectral similarity score (≥ 0.7) between the experimental and reference MS/MS spectra, as well as concordant retention time with an authentic standard. Level 2: Probable identification, supported by a moderate spectral match score (between 0.5 and 0.7) and consistent retention time with database records. Level 3: Putative identification based solely on matching MRM parameters (precursor ion, product ion, RT, declustering potential, and collision energy) without MS/MS spectral evidence; this level is considered to have lower reliability.

Quantitative analysis was conducted using a triple quadrupole mass spectrometer (AB Sciex 4500 QTRAP) in MRM mode. Characteristic ion pairs were selectively monitored for each metabolite, and signal intensities (in CPS) were recorded. Chromatographic peak integration and correction were performed using MultiQuant software (v3.0.3), and peak areas were used for relative quantification. To improve data quality, missing values were filled with one-fifth of the minimum value per metabolic row. Metabolites with a coefficient of variation (CV) ≥ 0.5 in quality control (QC) samples were excluded from further analysis.

### cDNA library construction

Total RNA was extracted from plant tissues using the Polysaccharide Polyphenol Plant Total RNA Extraction Kit (Tiangen Biochemical, Beijing, China). RNA integrity was assessed using the Bioanalyzer 2100 (Agilent Technologies, CA, USA). mRNA was purified from total RNA using Oligo(dT) magnetic beads. Using the SMARTer PCR cDNA Synthesis Kit (Takara Bio, Kusatsu, Japan), cDNA was synthesized with random hexamer primers. A sequencing library was constructed through sequential steps including end repair, A-tailing, adapter ligation, size selection, PCR amplification, and purification. The quality of the library was assessed by quantifying with Qubit and real-time PCR, and size distribution was analyzed using the Bioanalyzer. Finally, the library was sequenced on the PacBio Sequel platform (Pacific Biosciences, Menlo Park, CA, USA) at Novogene Bioinformatics Institute (Beijing, China).

### Transcriptome assembly and quantification

Full-length transcriptome sequencing was performed on the PacBio Sequel platform. Raw subreads were processed using the SMRTlink 11.0 software package to generate circular consensus sequences (CCS). CCS reads were classified as full-length non-chimeric (FLNC) transcripts based on the presence of 5’ and 3’ primers and poly(A) tails. FLNC transcripts were then clustered using the ICE algorithm to produce consensus isoforms. These consensus isoforms were subsequently polished with hq_quiver_min_accuracy 0.99, bin_by_primer false, bin_size_kb 1, qv_trim_5p 100, qv_trim_3p 30 [38].

For gene expression quantification, Illumina RNA-seq reads from each sample were independently mapped to this PacBio-derived reference transcriptome using Bowtie2 [39]. The abundance of each transcript was estimated and normalized to Fragments Per Kilobase of transcript per Million mapped reads (FPKM) values using RSEM software.

### Bioinformatics analysis

The processed full-length transcriptome data and RNA-seq assembled transcript were subjected to redundancy removal using CD-HIT software. These sequences were compared against seven databases: NR [40], Nt, Pfam, KOG/COG [41], Swiss-prot [42], KEGG [43], and GO [44], to conduct gene functions. The ANGEL software [45] was utilized for CDS prediction analysis. Transcripts were quantified and further transformed into FPKM values, which served as the expression levels across different samples.

### Identification, sequence analyses and phylogenetic of differential gene expression

Differential expression analysis was performed using the DESeq2 package (1.20.0) in R. DESeq2 employs statistical methods based on a negative binomial generalized linear model to determine differential expression in digital gene expression data [46]. The resulting P-value were adjusted for multiple testing using the Benjamini-Hochberg method to control the false discovery rate (FDR). Genes with |log₂(fold change)| ≥ 1 and an adjusted P-value (padj) < 0.05 were considered significantly differentially expressed.

Gene sequences of interest were retrieved from the full-length transcriptome annotation table. Multiple sequence alignment was performed using MEGA Ⅶ, and the longest sequence for each gene was selected. Reciprocal BLAST analysis was conducted between the full-length gene sequences and RNA-seq transcripts using BLAST 2.2.31 to confirm sequence homology and ensure sequence accuracy.

Phylogenetic relationships within the SSR and SMO gene families were reconstructed using the neighbor-joining (NJ) algorithm in MEGA Ⅶ (version 7.0.26). Bootstrap support values were calculated based on 1,000 replicates to assess the reliability of the phylogenetic tree.

### CYP450 Gene family identification and phylogenetic analysis

Transcripts annotated to CYP450 families were identified from the PFAM annotation file of the *F. taipaiensis* full-length transcriptome. The corresponding nucleotide sequences were retrieved from the polished high-quality consensus FASTA file using SeqKit. Open reading frames (ORFs) were predicted from these non-redundant sequences using ORFfinder, and any predicted amino acid amino acid sequences shorter than 300 residues were excluded. The resulting amino acid sequences were subsequently used as queries to perform a local BLASTP search against the reference cytochrome P450 database (http://www.p450.kvl.dk/blast.shtml). For each query, the top significant hit (E-value <1e-10) was retained for subsequent analysis. Based on the sequence similarity to these top hits, the amino acid sequences were renamed according to the standard CYP450 nomenclature and deduplicated. A maximum-likelihood phylogenetic tree was constructed from the final dataset using TreeBest (v1.9.2) under the JTT substitution model, with branch support assessed from 1000 bootstrap replicates. The resulting tree was visualized and annotated using iTOL (v6.7).

### RT-qPCR validation of differentially expressed

cDNA was synthesized from total RNA for RT-qPCR analysis. Gene-specific primers (designed using Primer Premier 5.0, Palo Alto, CA, USA) were used with the CFX96™ Real-Time PCR System (Bio-Rad, USA). Reactions (10 µl total volume) contained ChamQ SYBR qPCR Master Mix (Vazyme, Q311) according to manufacturer’s instructions, along with primers and cDNA template. FrtActin1 served as the reference gene for normalization. The thermal cycling protocol consisted of an initial denaturation at 94 °C for 3 min, followed by 40 cycles of 94 °C for 15 s, 55 °C for 15 s, and 72 °C for 20 s. Relative transcript levels of target genes were calculated using the 2^(-ΔΔCt) method. Primer sequences are listed in Table S1.

### Agrobacterium-mediated transient transformation of tobacco

The candidate gene-containing binary vector p35S-Cambia 1300-GFP was transformed into *A. tumefaciens* GV3101, and the engineered strain was resuspended to *OD*₆₀₀=0.6 with infiltration buffer (10 mmol・L⁻¹ MgCl₂, 10 mmol・L⁻¹ MES, 200 µmol・L⁻¹ acetosyringone) after culture, then statically incubated at 28 °C in the dark for 3 h. Thirty-day-old uniform tobacco plants were subjected to whole-plant vacuum infiltration (-0.1 MPa, 5 min) with the bacterial suspension, and leaves were collected at 7 dpi, freeze-dried and stored at -80 °C for further analysis.

### Extraction and detection of sterols

Sterol extraction and derivatization were performed as follows. Approximately 10 mg of vacuum-dried leaf powder was saponified with 1 mL of methanolic KOH (containing coprostanol as internal standard) at 75 °C for 1 h. After ethanol evaporation, the mixture was extracted with ethyl acetate, and the organic layer was collected and dried. The residue was derivatized with BSTFA-TMCS (99:1) at 70 °C for 30–40 min.

GC‑MS analysis was carried out on a Thermo ISQ‑LT system equipped with an HP‑5MS column. Separation used a temperature program (170 °C for 2 min, 5 °C/min ramp to 290 °C, hold 5 min) with helium as carrier gas (1.2 mL/min). Full-scan MS (m/z 60–650, 14 min solvent delay) was applied for detection. Sterols were quantified via the internal standard method and identified by matching retention times/mass spectra with authentic standards (isofucosterol was tentatively identified using published spectral data). A QC sample was analyzed every 10 injections to ensure data reliability [47–49].

## Results

### Morphological characteristics and alkaloid profiling

**Fig. 1:**
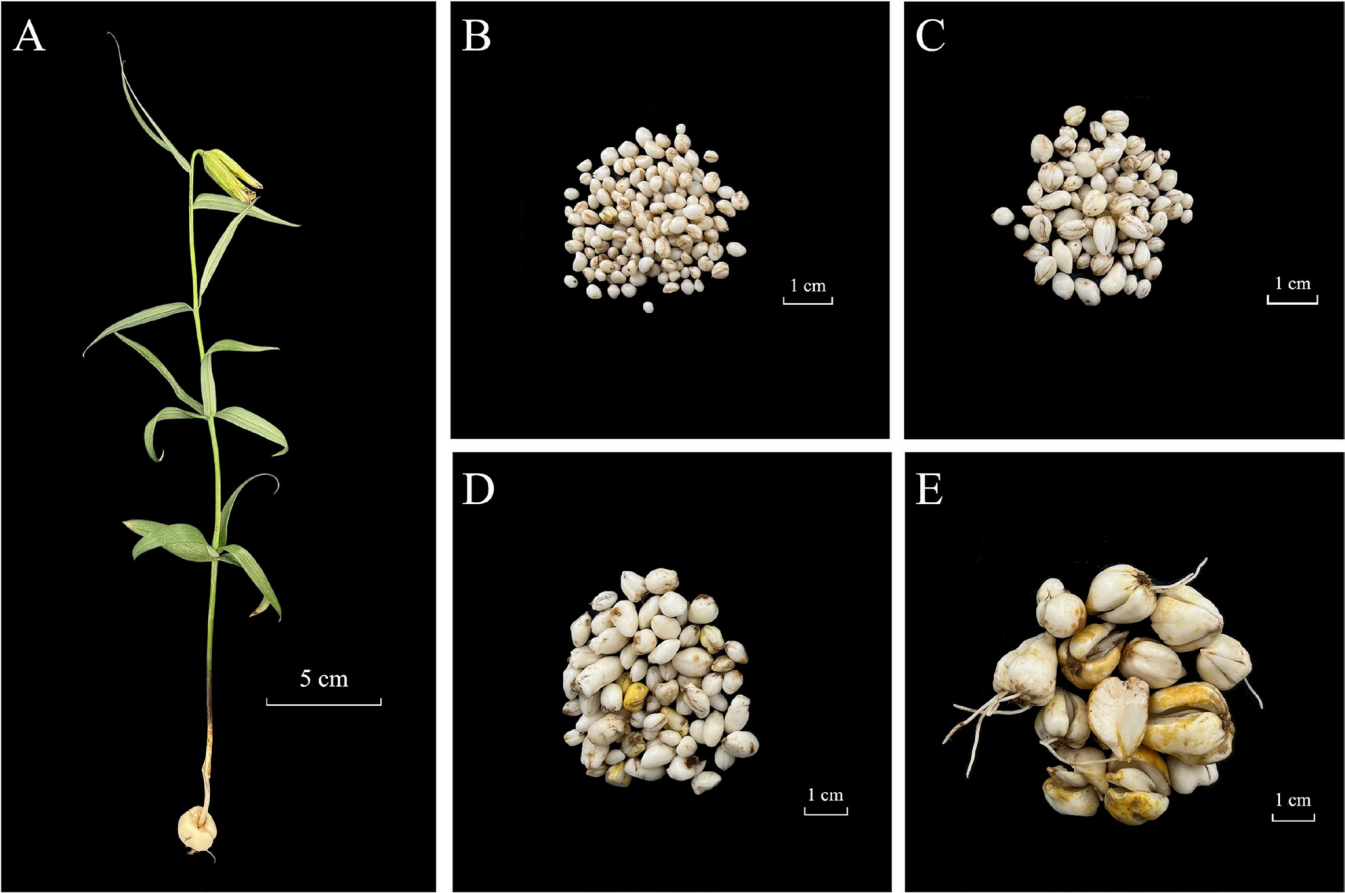
Morphological phenotypes of the medicinal bulb in F. taipaiensis. **A**. Whole plant morphology of 4-year-old F. taipaiensis. Bulb development of F. taipaiensis during consecutive growth years: **B**. 1-year-old bulb; **C**. 2-year-old bulb; **D**. 3-year-old bulb; **E**. 4-year-old bulb (used as medicinal material). Scale bars: A = 5 cm; B–E = 1 cm

The bulb, which is the primary medicinal organ of *F. taipaiensis* (Fig. 1), has an oblate morphology and consists of two scales measuring 1–1.5 cm in diameter (Fig. 1; Fig. S1). To characterize alkaloid accumulation patterns, untargeted UPLC-MS/MS-based metabolomics was performed across bulb and leaf tissues, revealing distinct tissue-specific profiles.

**Fig. 2:**
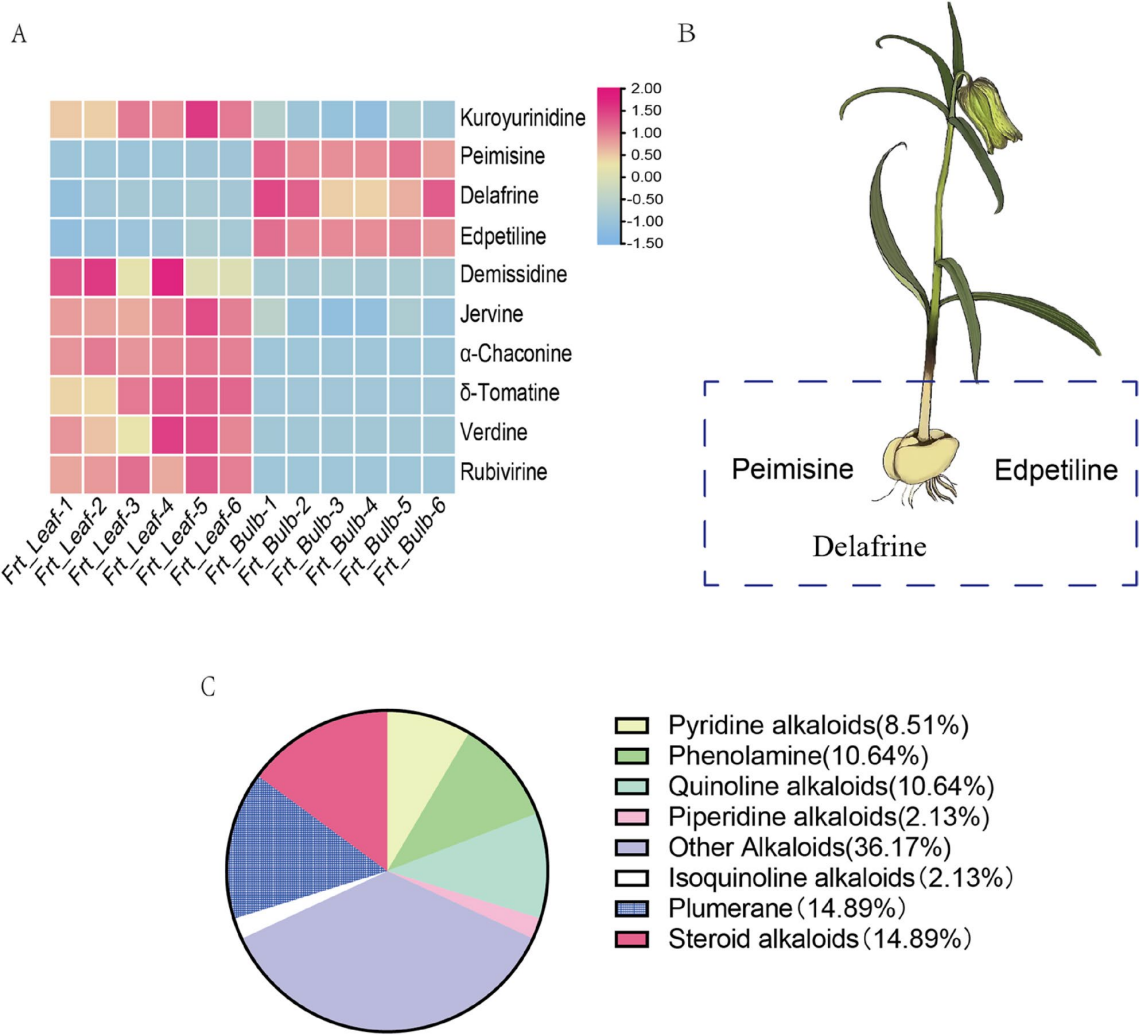
Analysis of differently accumulated alkaloids in various tissues of F. taipaiensis. **A**. Heatmap of identified steroidal alkaloid metabolites, with color intensity reflecting relative expression levels. **B**. Enriched steroidal alkaloid in bulb. **C**. Pie chart illustrating the compositional ratio of down regulated alkaloid species in root tissues

Among the 242 annotated alkaloid features, 21 were significantly upregulated in bulbs (fold change [FC] ≥ 2, variable importance in projection [VIP] > 1, false discovery rate [FDR] < 0.05; Files S1, S2). Of which, three steroidal alkaloids—peimisine, edpetiline, and delafrine—were identified with level 1 or 2 confidence (Fig. 2A, B). Specifically, peimisine, a principal bioactive steroidal alkaloid linked to the well-documented medicinal properties of *Fritillaria* species, exhibited high abundance in bulb tissue. Conversely, 146 alkaloids were downregulated in bulbs, 47 of which were confidently identified (Level 1 or 2) (Fig. 2A; File S1). This downregulated subset included diverse structural classes, such as quinoline alkaloids, plumerane-type alkaloids, and pyridine alkaloids (Fig. 2C)

### Transcriptomic landscape of bulb-specific gene expression

**Fig. 3:**
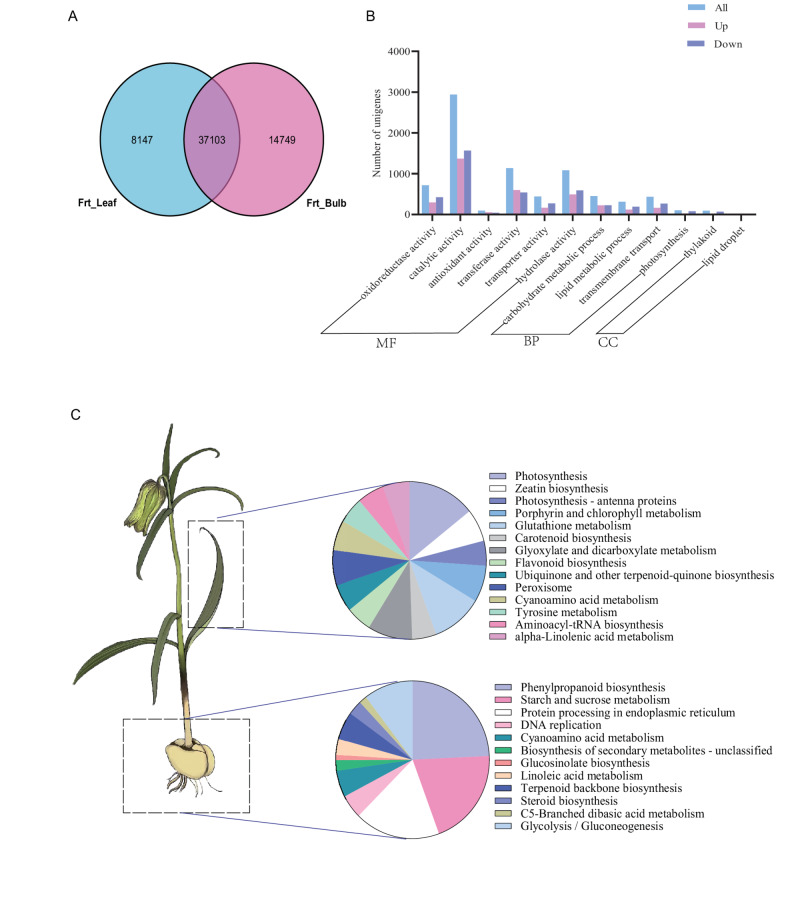
Transcriptional variation between the bulb and leaf of F. taipaiensis. **A**. A Venn diagram showing the comparison of expressed genes between the bulb and leaf of F. taipaiensis. **B**. GO enrichment analysis of differentially expressed genes (DEGs) in F. taipaiensis. MF: Molecular Function; BP: Biological Process; CC: Cellular Component. **C**. KEGG pathway enrichment analysis of DEGs in F. taipaiensis reveals the enriched metabolic pathways

Total RNA was extracted from bulb and leaf tissues of four-year-old *F. taipaiensis* plants (three biological replicates per group; *n* = 3) and sequenced. High inter-replicate correlations (Pearson’s r² > 0.88; Fig. S3A) and substantial gene expression overlap (73–78%; Fig. S3B) confirmed the robustness of the data. Differential expression analysis identified 11,750 differentially expressed genes (DEGs) between tissues, comprising 5,879 bulb-upregulated and 5,871 bulb-downregulated genes (Fig. 3A, Fig. S3C).

Gene Ontology (GO) enrichment analysis categorized differentially expressed genes (DEGs) into 12 functional classes, with molecular functions dominating, including “catalytic activity,” “oxidoreductase activity,” “transferase activity,” and “hydrolase activity” (Fig. 3B). KEGG pathway analysis revealed that bulb-upregulated DEGs were significantly enriched in “phenylpropanoid biosynthesis,” “starch/sucrose metabolism,” “terpenoid backbone biosynthesis,” and “steroid biosynthesis” (Fig. 3C; File S4). Notably, genes involved in the phenylpropanoid pathway, including *cinnamate-4-hydroxylase* (*CYP73A*), *cinnamyl alcohol dehydrogenase* (*CAD*), *4-coumarate-CoA ligase* (*4CL*), and *phenylalanine ammonia-lyase* (*PAL*), were coordinately upregulated (Fig. S4).

### Molecular basis of steroidal alkaloid biosynthesis

**Fig. 4:**
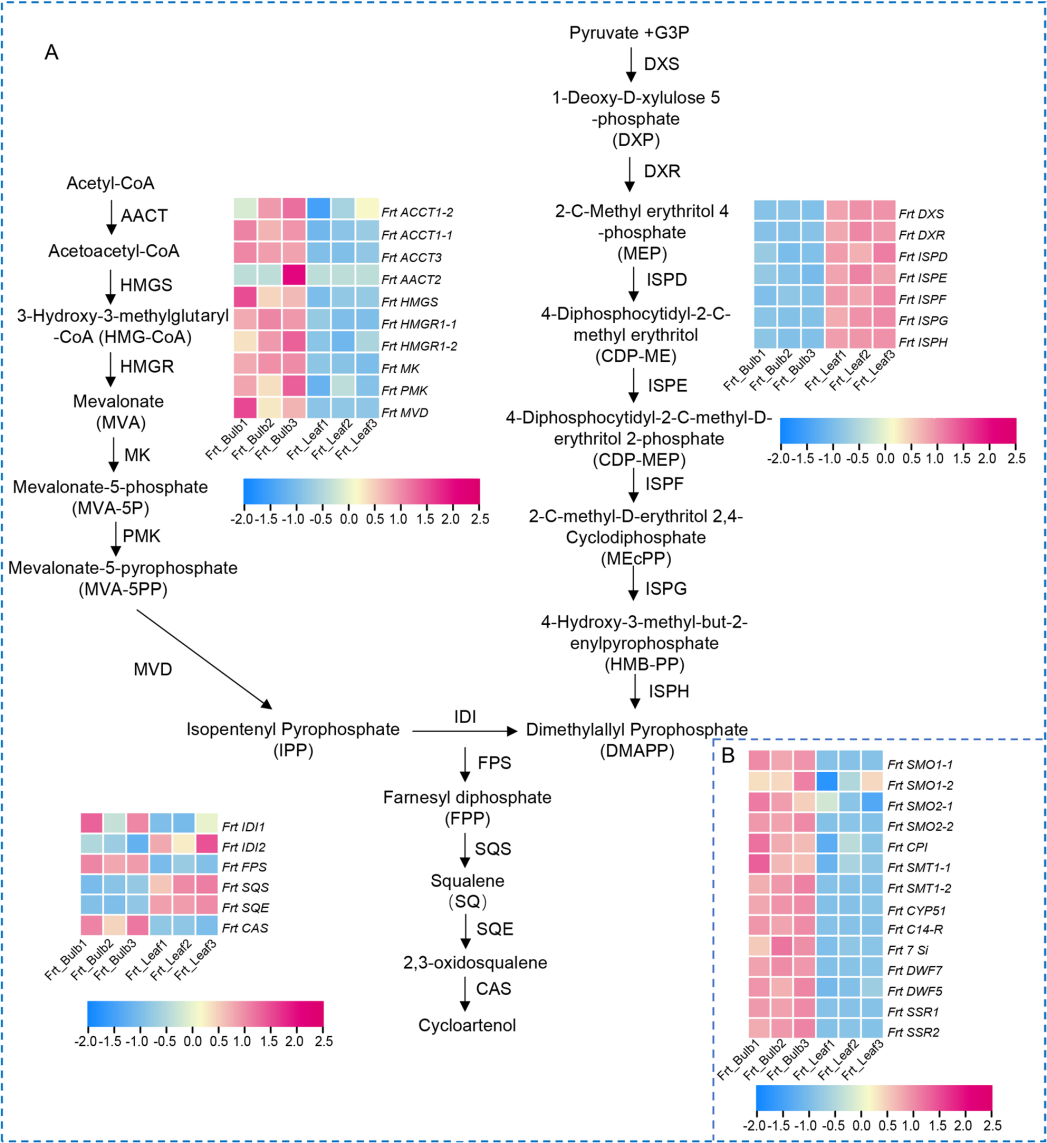
DEGs in sterol skeleton biosynthesis in F. taipaiensis. **A**. Expression profiles of DEGs in bulb and leaf tissues within cycloartenol biosynthetic pathway. Horizontal rows represent individual UniGenes, and vertical columns represent each sampled point. Gene expression data were normalized using the Z-score method, with color intensity reflecting relative expression levels, red indicates high expressed genes and blue indicates low expressed genes.** B**. Expression profiles of additional genes involved in sterol skeleton biosynthesis. Genes are listed from top to bottom: *FrtSMO1* (sterol methyltransferase 1), *FrtSMO1-1*, *FrtSMO2*, *FrtSMO2-2*, *FrtCPI *(cyclopropane fatty acid synthase), *FrtSMT1* (sterol methyltransferase 1), *FrtSMT1-1*, *FrtCYP51* (cytochrome P450 51), *FrtC14R* (farnesyl kinase), *Frt8,7-SI* (Δ8-Δ7 isomerase), *FrtDWF7* (de-etiolated 2), *FrtDWF5* (brassinosteroid synthase), *FrtSSR1* (sterol side-chain reductase 1 isoform 1), *Frt SSR2*

The biosynthetic network of cycloartenol, a key intermediate in steroidal alkaloid formation, is likely subject to intricate tissue-specific regulation in *F. taipaiensis*. Transcriptomic analysis revealed a significantly higher expression of MVA pathway genes in bulbs relative to leaves (Fig. 4A, Fig. S5). In contrast, genes involved in MEP pathway were predominantly expressed in leaves.​ Differential expression of isomerase genes was also observed: *IDI1* transcripts were predominantly detected in bulbs, whereas *IDI2* showed higher expression in leaves, suggesting tissue-specific utilization of isoprenoid precursors. Consistent with this pattern, genes encoding *FPS* and *CAS* were upregulated in bulbs. Conversely, *SQS* and *SQE* exhibited higher expression in leaves. Notably, key downstream modification enzymes were markedly upregulated in bulbs. These included *SMO1-1*, *SMT1*, and *CYP51* (Fig. 4B; File S5). These findings indicate a pronounced divergence in metabolic flux after cycloartenol formation between the two tissues.

To further investigate how these DEGs relate to steroidal alkaloid accumulation, we conducted an integrated transcriptome-metabolome correlation analysis. The results revealed significant correlations between the expression of MVA pathway genes and key cholesterol biosynthetic enzymes (including *FPS*, *CAS*, *CYP51*, *SMO1-1*, and *SSR1*) and the levels of final steroidal alkaloid metabolites, suggesting their critical roles in the biosynthetic pathway (Fig. S6).

### Functional diversification of sterol C-4 demethylase (SMO) in *F. taipaiensis*

**Fig. 5:**
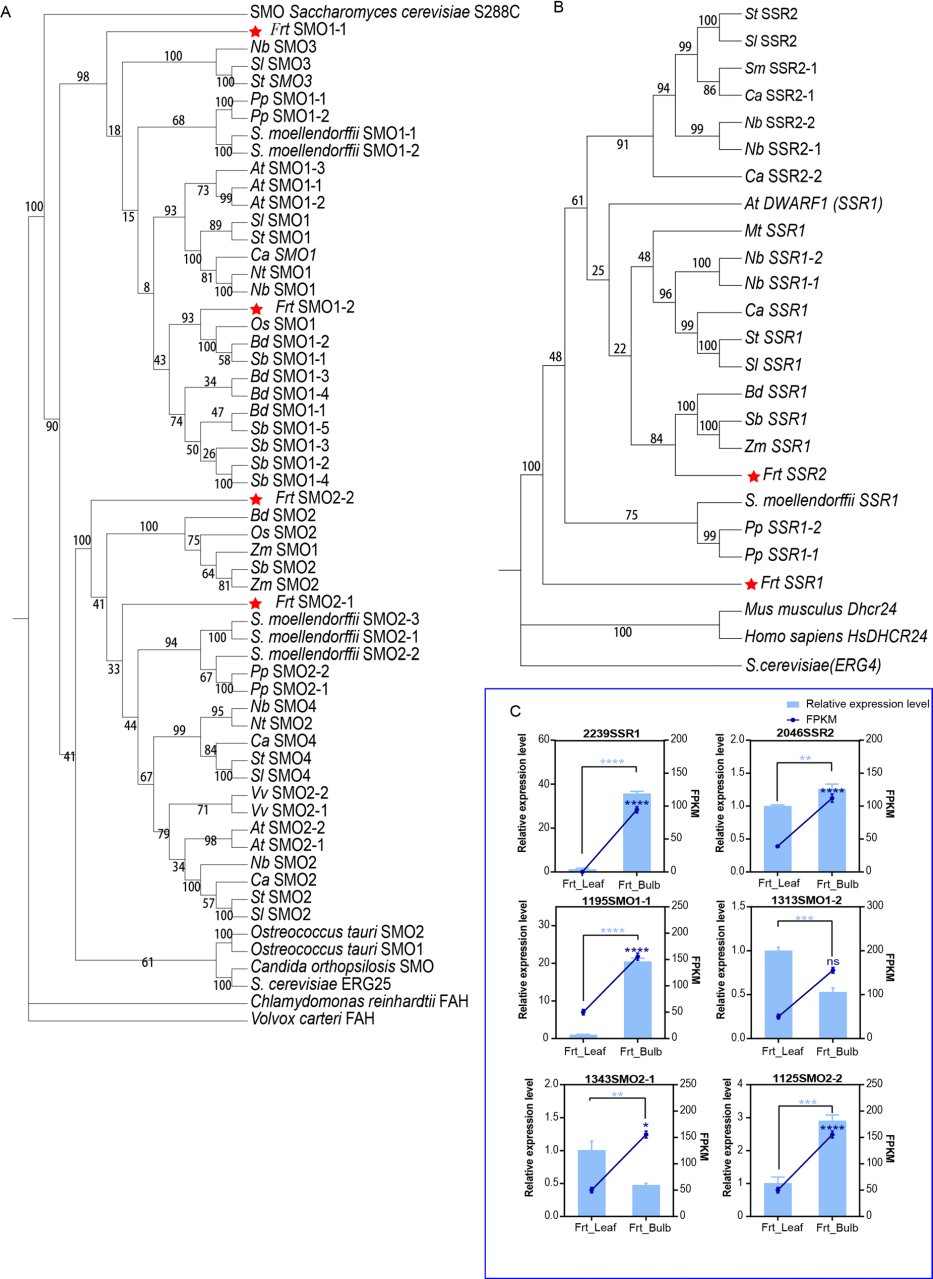
Phylogenetic analysis of SMO and SSR, and validation of gene expression levels. **A-B**. Phylogenetic relationships of SMO (A) and SSR (B) proteins across diverse plant species. Tobacco (Nt), *N.Benthamiana* (Nb), *Capsicum annuum* (Ca), tomato (Sl), potato(St), *Arabidopsis thaliana* (At), *Physcomitrella patens* (Pp), rice (Os), *Brachypodium distachyon* (Bd), *Sorghum bicolor* (Sb), *Vitis vinifera* (Vv), Maize (Zm), *Medicago truncatula *(Mt), *Selaginella moellendorffii* (*S.moellendorffii*), Eggplant (Sm), *Saccharomyces cerevisiae* (*S.cerevisiae*). The evolutionary history was inferred using the Neighbor-Joining method in MEGA7. **C**. qRT-PCR validation of gene expression level in the transcriptome. The bar chart represents the qRT-PCR, and the line chart represents the FPKM. ns means no significant difference, *P < 0.05, **P < 0.01, ***P < 0.001, ****P < 0.0001, (t-test)

Functional diversification of SMO was investigated in *F. taipaiensis*. Full-length sequencing identified four SMO isoforms, including two SMO1 and two SMO2 variants, indicating lineage-specific gene duplication. Phylogenetic analysis revealed distinct evolutionary pathways. FrtSMO1-2 clustered with monocot SMO1 homologs, whereas FrtSMO1-1 was placed outside the well-defined SMO1/SMO3 clade. Similarly, FrtSMO2-2 grouped with eudicot SMO2/SMO4 homologs, while FrtSMO2-1 was placed outside the well-defined SMO2/SMO4 clade, suggesting potential functional divergence (Fig. 5A).

Quantitative RT-PCR (qPCR) analysis revealed significant tissue-specific expression differences. *FrtSMO1-1* exhibited a striking higher expression in bulbs compared to leaves (Fig. 5C), consistent with the tissue-specific enrichment of SMO3/SMO4 isoforms in alkaloid-accumulating tissues of solanaceous species [21]. In contrast, *FrtSMO1-2* displayed only a slightly enrichment in leaves. *FrtSMO2-2* expression was highly induced in bulbs relative to leaves, while *FrtSMO2-1* showed a slightly accumulation in leaf enrichment (Fig. 5C).

These divergent expression patterns suggest functional specialization among SMO isoforms. FrtSMO1-1 likely facilitates C-4 demethylation to support alkaloid-specialized cholesterol flux, whereas FrtSMO1-2 may sustain constitutive phytosterol biosynthesis. Conversely, FrtSMO2-2 is hypothesized to enhance the production of cholesterol precursors, and FrtSMO2-1 likely contributes to basal sterol metabolism.

### Evolutionary divergence of sterol reductases in *F. taipaiensis*

Full-length transcriptome sequencing also resolved two 24-dehydrocholesterol reductase homologs in *F. taipaiensis*, designated *FrtSSR1* and *FrtSSR2*. Phylogenetic analysis placed FrtSSR2 within a clade of monocot DWF1 homologs, suggesting a conserved role in sterol biosynthesis among monocots. In contrast, FrtSSR1 formed a distinct clade sister to the canonical plant SSRs (Fig. 5B), indicating early divergence from canonical sterol reductases.

**Fig. 6:**
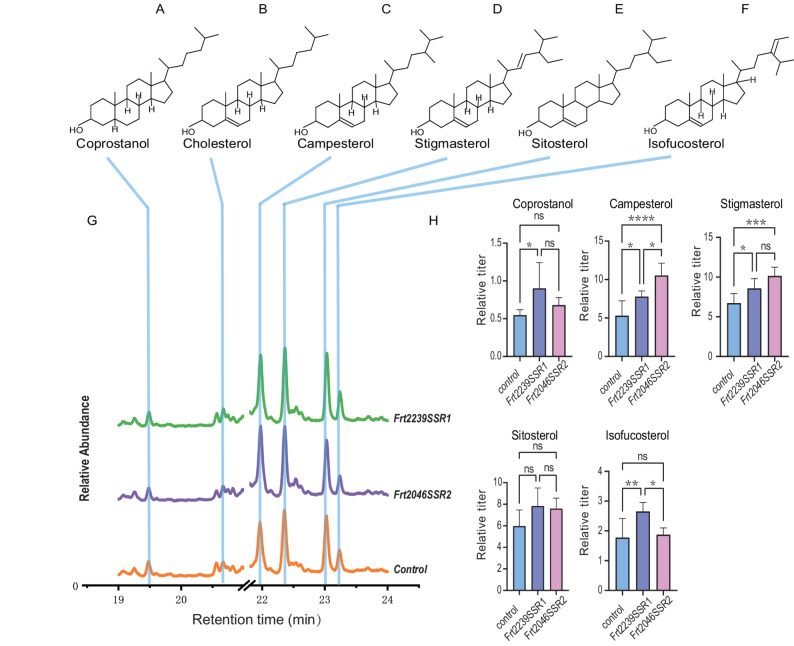
Functional characterization of *FrtSSR1* and *FrtSSR2 *by transient expression in Nicotiana benthamiana. ( **A-F**. Chemical structures of the sterols detected in this assay: **A**. cholestanol, **B**. cholesterol, **C**. campesterol, **D**. stigmasterol, **E**. sitosterol, and **F**. isofucosterol. G. Representative GC-MS total ion current (TIC) profiles of sterol extracts from Nicotiana benthamiana leaves transiently expressing empty vector (Control, orange), *FrtSSR2* (purple), or *FrtSSR1* (green). The x-axis indicates retention time (min), and the y-axis indicates relative abundance of sterol ions. **H**. Quantification of relative sterol abundance under each treatment. Data are presented as mean ± SE. Statistical significance was determined by one-way ANOVA, ns means no significant difference, *P < 0.05, **P < 0.01, ***P < 0.001, ****P < 0.0001

qPCR revealed that *FrtSSR1* expression in bulbs was striking higher than in leaves (Fig. 5C), a pattern reminiscent of *SlSSR2* in Solanaceae, which is involved in cholesterol-dependent alkaloid biosynthesis [23]. This strongly implies a functional role for FrtSSR1 in catalyzing Δ²⁴(²⁵)-sterol reduction—a key step in bulb-specific steroidal alkaloid production. In contrast, *FrtSSR2* exhibited only minimal expression variation between tissues (1.2-fold), supporting the hypothesis that it contributes primarily to basal sterol homeostasis through Δ²⁴(²⁸)-bond reduction, consistent with its phylogenetically conserved function. To experimentally validate these functional predictions, we performed transient expression assays of *FrtSSR1* and *FrtSSR2* in *Nicotiana benthamiana*. Gas chromatography-mass spectrometry (GC-MS) analysis showed that transient expression of *FrtSSR1* significantly increased cholesterol levels—the direct precursor of steroidal alkaloids—whereas *FrtSSR2* expression elevated levels of campesterol and stigmasterol, which are essential for membrane sterol homeostasis. These finding confirm the functional divergence of the two paralogs (Fig. 6). While final steroidal alkaloids were not detected in this heterologous expression system, the distinct sterol precursor profiles further validate the proposed subfunctionalization of FrtSSR1 and FrtSSR2 following gene duplication.

### Functional diversification of cytochrome P450 monooxygenases in *F. taipaiensis*

**Fig. 7:**
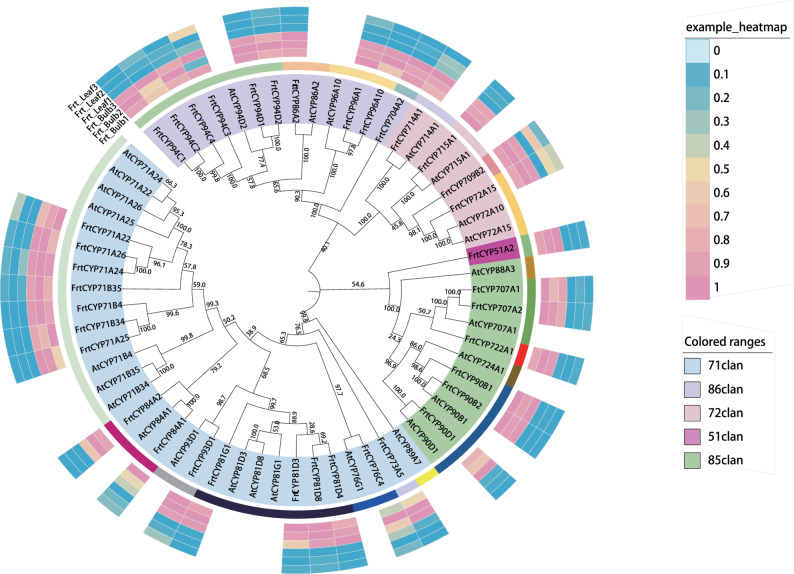
Phylogenetic relationship and expression profiles of differentially expressed CYP450 genes in *F. taipaiensis*. The circular phylogenetic tree illustrates the evolutionary relationships of CYP450 genes that are differentially expressed between bulbs and leaves of* F. taipaiensis*, inferred based on sequence homology. Genes are classified into five major clans (Clan 51, 71, 72, 85, and 86) according to the Nelson classification system, with each clan color-coded as follows: Clan 71, light blue; Clan 86, lavender; Clan 72, light pink; Clan 51, magenta; Clan 85, green. The inner ring adjacent to gene labels indicates family-level classification within each clan, distinguished by distinct colors. The outermost ring displays normalized expression levels of these CYP450 genes in bulbs and leaves, with each column representing a tissue type and each row corresponding to an individual gene. Expression values are visualized using a color gradient ranging from blue (low expression, 0) to pink (high expression, 1)

Our transcriptome analysis revealed distinct tissue-specific induction patterns of P450 genes, suggesting their functional involvement in compartmentalized metabolic accumulation (Fig. 7, S7). For instance, *CYP51* was upregulated 7.8-fold in bulbs, consistent with its conserved role in sterol biosynthesis, potentially serving as a scaffold for steroidal alkaloid diversification. Within Clan 71, multiple families showed bulb induction, including CYP71 (*CYP71B4/34/35* and *CYP71A22/24/25/26*), CYP81 (*CYP81D3/8* and *CYP81G1*), and CYP76 (*CYP76C4*). Based on homology to characterized P450s from other alkaloid-producing plants such as *Catharanthus roseus* and *Papaver somniferum*, these enzymes are putative candidates for catalyzing oxidative modifications—such as hydroxylation, epoxidation, or skeletal rearrangements—on the steroidal skeleton.

In Clan 86, induced isoforms including *CYP94C1-4*, *CYP94D1/2*, and *CYP86A2*, often associated with fatty acid ω-hydroxylation, suggests a possible role in generating lipid-derived precursors or signaling molecules that could indirectly influence alkaloid biosynthesis pathways. Similarly, elevated expression of CYP96 family members might modulate jasmonic acid signaling, potentially creating a transcriptional environment conducive to alkaloid accumulation, as observed in other medicinal plants. Within Clan 85, the induction of *CYP707A1/2* a link between abscisic acid catabolism and bulb development, potentially connecting stress responses to alkaloid synthesis. The upregulation of *CYP90B1/2* and *CYP90D1*, known for their role in brassinosteroid biosynthesis via C-22/C-23 hydroxylation, raises the intriguing possibility that these enzymes might also exhibit catalytic versatility towards steroidal alkaloid precursors.

Integrated transcriptome‑metabolome correlation analysis further supported the functional relevance of these P450s, revealing significant associations between their expression profiles and the accumulation of specific steroidal alkaloids (Fig. S6), suggesting their critical and diversified roles in the biosynthetic pathway.

## Discussion

### The biosynthesis and accumulation of alkaloids in *F. taipaiensis* exhibit organ-specific patterns

Our integrated metabolomic and transcriptomic analyses definitively identify the bulb as the primary site for the accumulation of pharmacologically relevant steroidal alkaloids, including peimisine, edpetiline, and delafrine. In contrast, leaves accumulate a distinct set of alkaloids, likely serving ecological defense roles. This organ-specific metabolic division is underpinned by complementary transcriptional programs. The bulb-specific commitment to steroidal alkaloid biosynthesis is evidenced by the coordinated upregulation of the MVA pathway and key downstream enzyme genes (e.g., FPS, CAS). This is coupled with a pronounced enhancement in cholesterol flux, facilitated by the specific induction of *SMO1-1* and *SSR1*. This functional specialization between organs enables efficient precursor partitioning: the cytosolic MVA pathway in bulbs utilizes acetyl-CoA from carbohydrate metabolism, while the plastidial MEP pathway in leaves relies on photosynthetic metabolites [50, 51]. Such compartmentalization likely reflects an evolutionary optimization for resource allocation, positioning the bulb as a dedicated sink for the synthesis and storage of high-value medicinal compounds [52, 53].

### Evolutionary divergence of key enzymes orchestrates biosynthetic specialization

The functional specialization between bulb and leaf tissues is further elucidated by evolutionary innovations in key sterol-modifying enzymes. Gene duplication and subsequent neofunctionalization within the SMO and SSR families have enabled tissue-specific regulation of cholesterol precursor flux [21, 23]. we observed lineage-specific expansion resulting in four SMO isoforms and two SSR homologs. Crucially, *FrtSMO1-1* and *FrtSSR1* exhibit pronounced bulb-specific expression, suggesting their dedicated roles in channeling metabolic flux toward steroidal alkaloid biosynthesis. Phylogenetic analyses indicate that these bulb-specific isoforms occupy early-diverging positions, implying subfunctionalization events that fine-tune sterol metabolism in a tissue-specific manner. Our functional validation via transient expression in *Nicotiana benthamiana* directly supports this specialization: *FrtSSR1* expression significantly increased cholesterol, whereas *FrtSSR2* elevated core membrane sterols. Even in the absence of detectable final steroidal alkaloids in this heterologous system, the distinct sterol precursor profiles further validate the subfunctionalization of these paralogs following gene duplication. These findings collectively exemplify how gene family evolution can orchestrate metabolic compartmentalization, enabling the optimization of specialized metabolite production in target tissues, which is a key consideration for targeted metabolic engineering efforts.

### P450-mediated structural diversification and metabolic pathway crosstalk

The tissue-specific induction of numerous P450 genes in bulbs may suggest a potential role in generating alkaloid structural diversity and facilitating metabolic coordination, though this remains unconfirmed by direct experimental evidence. The upregulation of Clan 71 P450s could implies their possible involvement in oxidative modifications of the steroidal skeleton, an inference tentatively supported by homologs in other medicinal plants like *Catharanthus roseus* [54]. The concurrent upregulation of phenylpropanoid pathway genes may hint at potential metabolic crosstalk, which might potentially supply essential cofactors or energy to support P450-catalyzed reactions,, though this crosstalk has not been experimentally validated [55]. The induction of Clan 86 P450s, often associated with fatty acid metabolism, raises the possibility that these isoforms could play a role in generating lipid-derived intermediates for steroidal side-chain modification [56]. Furthermore, the co-upregulation of brassinosteroid-related *CYP90B1/2* and *CYP90D1* suggest a potential catalytic versatility towards steroidal alkaloid precursors [57–59]. While these functional assignments remain speculative in the absence of direct functional validation, however, they may provide a preliminary foundation for future hypothesis-driven research. The identification of these candidate CYP450s is a significant step forward, as they represent high-value targets for engineering the production of specific, potentially more potent or novel, steroidal alkaloid derivatives.

## Conclusions

In summary, this study elucidates the molecular and metabolic basis of steroidal alkaloid biosynthesis in *F. taipaiensis* through integrated multi-omics analysis. Three major conclusions emerge: (1) Tissue-specific metabolic specialization is suggested to underlie alkaloid accumulation, with bulbs tentatively inferred to activate the MVA pathway and candidate key cholesterol biosynthetic genes (FPS, CAS, CYP51, SMO1-1, FrtSSR1), which may facilitate the production of steroidal precursors. (2) Gene duplication and subfunctionalization may drive functional divergence, with bulb-preferentially gene expressed isoforms (e.g., *FrtSSR1*, *FrtSMO1-1*) potentially involved in alkaloid biosynthesis, while their paralogs likely responsible for core sterol homeostasis. (3) Multiple P450 families are suggested to be implicated in the structural diversification and metabolic coordination of steroid alkaloids, which may present a potential genetic resource for further exploring alkaloid diversification.

## Supplementary Information

Supplementary Material 1.

Supplementary Material 2.

Supplementary Material 3.

Supplementary Material 4: Table S1. Primer information.

Supplementary Material 5.

Supplementary Material 6.

Supplementary Material 7: The following supplementary data are available. Fig. S1. The phenotype of *F. taipaiensis*; Fig. S2. Multivariate analysis of metabolomic data from bulb and leaf tissues of *F. taipaiensis*; Fig.S3. Correlation and differential gene expression analysis in *F. taipaiensis*; Fig. S4. Comparison of gene expression levels in the phenylpropanoid pathway (by FPKM values) between bulbs and leaves of the *F. taipaiensis*; Fig.S5. The qRT-PCR validation confirms the gene expression levels derived from the transcriptome analysis; Fig.S6. Pearson correlation plot of DEGs putatively involved in the steroidal alkaloid biosynthetic pathway and steroidal alkaloid metabolites in *F.taipaiensis*. Fig.S7. Comparison of gene expression levels of CYP450 gene family in *F. taipaiensis* (by FPKM values) between bulbs and leaves; File S1 Information Table of All Metabolites in *F. taipaiensis* by UPLC-MS-MS; File S2 Differential Metabolites Profiling between Bulb and Leaf Tissues of *F. taipaiensis*; File S3 Summary of Transcriptomic Sample Quality Metrics for *F. taipaiensis*; File S4 KEGG enrichment of DEGs in leaf and bulb tissues of *F. taipaiensis*;File S5 Table of Enzyme Genes Involved in the Steroidal Alkaloid Biosynthesis Pathway of *F. taipaiensis.*

## Data Availability

The data or material of this study are available from the corresponding author, H.Y.R. and Y. Q., upon reasonable request.

## References

[1] Chen T, Zhong F, Yao C, Chen J, Xiang Y, Dong J, Yan Z, Ma Y. A Systematic Review on Traditional Uses, Sources, Phytochemistry, Pharmacology, Pharmacokinetics, and Toxicity of Fritillariae Cirrhosae Bulbus. Evid Based Complement Alternat Med. 2020;2020:1536534. 10.1155/2020/1536534 PMC7676930 33273948

[2] Wu F, Tian M, Sun Y, Wu C, Liu X. Efficacy, chemical composition, and pharmacological effects of herbal drugs derived from Fritillaria cirrhosa D. Don and Fritillaria thunbergii Miq. Front Pharmacol. 2022;13:985935. 36532788 10.3389/fphar.2022.985935 PMC9748432

[3] Kumar P, Partap M, Ashrita, Rana D, Kumar P, Warghat AR. Metabolite and expression profiling of steroidal alkaloids in wild tissues compared to bulb derived in vitro cultures of Fritillaria roylei – High value critically endangered Himalayan medicinal herb. Ind Crops Prod. 2020;145:111945.

[4] Wang X, Luo H-m, Wei X-m, Cao P, Gao Z-t, Han J-p. Transcriptome analysis provides insights into key gene(s) involved in steroidal alkaloid biosynthesis in the medicinally important herb Fritillaria taipaiensis. In.: Research Square; 2019.

[5] Guo K, Chen J, Niu Y, Lin X. Full-Length Transcriptome Sequencing Provides Insights into Flavonoid Biosynthesis in Fritillaria hupehensis. Life. 2021;11(4):287. 33800612 10.3390/life11040287 PMC8066755

[6] Wu X, Chan S-w, Ma J, Li P, Shaw P-c, Lin G. Investigation of association of chemical profiles with the tracheobronchial relaxant activity of Chinese medicinal herb Beimu derived from various Fritillaria species. J Ethnopharmacol. 2018;210:39–46. 28842340 10.1016/j.jep.2017.08.027

[7] Wong EL, Sung RY, Leung TF, Wong YO, Li AM, Cheung KL, Wong CK, Fok TF, Leung PC. Randomized, double-blind, placebo-controlled trial of herbal therapy for children with asthma. J Altern Complement Med. 2009;15(10):1091–7. 19821718 10.1089/acm.2008.0626

[8] Wang D, Chen X, Atanasov AG, Yi X, Wang S. Plant Resource Availability of Medicinal Fritillaria Species in Traditional Producing Regions in Qinghai-Tibet Plateau. Front Pharmacol. 2017;8:502. 10.3389/fphar.2017.00502 PMC5545572 28824427

[9] Mathela M, Kumar A, Sharma M, Goraya GS. Hue and cry for Fritillaria cirrhosa D.Don, a threatened medicinal plant in the Western Himalaya. Discover Sustain. 2021;2(1):38.

[10] Lu Q, Li R, Liao J, Hu Y, Gao Y, Wang M, Li J, Zhao Q. Integrative analysis of the steroidal alkaloids distribution and biosynthesis of bulbs Fritillariae Cirrhosae through metabolome and transcriptome analyses. BMC Genomics. 2022;23(1):511. 35836113 10.1186/s12864-022-08724-0 PMC9284883

[11] Duan Y, Wu J, Wang F, Zhang K, Guo X, Tang T, Mu S, You J, Guo J. Transcriptomic and metabolomic analyses provide new insights into the appropriate harvest period in regenerated bulbs of Fritillaria hupehensis. Front Plant Sci. 2023;14:1132936. 36875619 10.3389/fpls.2023.1132936 PMC9975545

[12] Qu A, Wu Q, Su J, Li C, Yang L, Wang Z, Wang Z, Li Z, Ruan X, Zhao Y, et al. A Review on the Composition and Biosynthesis of Alkaloids and on the Taxonomy, Domestication, and Cultivation of Medicinal Fritillaria Species. Agronomy. 2022;12(8):1844.

[13] Qi P, Zhang Y, Zhao C, Sun L, Bai R, Wang L, Sun C. Research progress on biological regulation and biosynthesis of isosteroid alkaloids in Fritillaria. Plant Growth Regul. 2023;101(3):599–615.

[14] Liu S, Yang T, Ming TW, Gaun TKW, Zhou T, Wang S, Ye B. Isosteroid alkaloids with different chemical structures from Fritillariae cirrhosae bulbus alleviate LPS-induced inflammatory response in RAW 264.7 cells by MAPK signaling pathway. Int Immunopharmacol. 2020;78:106047. 31816576 10.1016/j.intimp.2019.106047

[15] Vranová E, Coman D, Gruissem W. Network Analysis of the MVA and MEP Pathways for Isoprenoid Synthesis. Annu Rev Plant Biol. 2013;64(64, 2013):665–700. 23451776 10.1146/annurev-arplant-050312-120116

[16] Liu Y, Zhou J, Hu T, Lu Y, Gao L, Tu L, Gao J, Huang L, Gao W. Identification and functional characterization of squalene epoxidases and oxidosqualene cyclases from Tripterygium wilfordii. Plant Cell Rep. 2020;39(3):409–18. 31838574 10.1007/s00299-019-02499-7

[17] Dong H, Qi X. Biosynthesis of triterpenoids in plants: Pathways, regulation, and biological functions. Curr Opin Plant Biol. 2025;85:102701. 40112428 10.1016/j.pbi.2025.102701

[18] Milner SE, Brunton NP, Jones PW, O’ Brien NM, Collins SG, Maguire AR. Bioactivities of Glycoalkaloids and Their Aglycones from Solanum Species. J Agric Food Chem. 2011;59(8):3454–84. 21401040 10.1021/jf200439q

[19] Dinan L. Phytoecdysteroids: biological aspects. Phytochemistry. 2001;57(3):325–39. 11393511 10.1016/s0031-9422(01)00078-4

[20] Cárdenas PD, Sonawane PD, Heinig U, Bocobza SE, Burdman S, Aharoni A. The bitter side of the nightshades: Genomics drives discovery in Solanaceae steroidal alkaloid metabolism. Phytochemistry. 2015;113:24–32. 25556315 10.1016/j.phytochem.2014.12.010

[21] Sonawane PD, Pollier J, Panda S, Szymanski J, Massalha H, Yona M, Unger T, Malitsky S, Arendt P, Pauwels L, et al. Plant cholesterol biosynthetic pathway overlaps with phytosterol metabolism. Nat Plants. 2016;3(1):16205. 28005066 10.1038/nplants.2016.205

[22] Bao X, Zhu Y, Li G, Liu L. Regulation of storage organ formation by long-distance tuberigen signals in potato. Hortic Res. 2025;12(4):360. 10.1093/hr/uhae360. 10.1093/hr/uhae360 PMC11894528 40070401

[23] Sawai S, Ohyama K, Yasumoto S, Seki H, Sakuma T, Yamamoto T, Takebayashi Y, Kojima M, Sakakibara H, Aoki T, et al. Sterol side chain reductase 2 is a key enzyme in the biosynthesis of cholesterol, the common precursor of toxic steroidal glycoalkaloids in potato. Plant Cell. 2014;26(9):3763–74. 25217510 10.1105/tpc.114.130096 PMC4213163

[24] Ziegler J, Facchini PJ. Alkaloid Biosynthesis: Metabolism and Trafficking. Annu Rev Plant Biol. 2008;59(59):735–69. 18251710 10.1146/annurev.arplant.59.032607.092730

[25] Li R, Xiao M, Li J, Zhao Q, Wang M, Zhu Z. Transcriptome Analysis of CYP450 Family Members in Fritillaria cirrhosa D. Don and Profiling of Key CYP450s Related to Isosteroidal Alkaloid Biosynthesis. Genes (Basel) 2023;14(1):219. 10.3390/genes14010219 PMC9859280 36672960

[26] Christ B, Xu C, Xu M, Li F-S, Wada N, Mitchell AJ, Han X-L, Wen M-L, Fujita M, Weng J-K. Repeated evolution of cytochrome P450-mediated spiroketal steroid biosynthesis in plants. Nat Commun. 2019;10(1):3206. 31324795 10.1038/s41467-019-11286-7 PMC6642093

[27] Zhao Y, Wu Z, Li J, Qi Y, Zhang X, Shen C. The key role of cytochrome P450s in the biosynthesis of plant derived natural products. Plant Physiol Biochem. 2025;222:109695. 40015195 10.1016/j.plaphy.2025.109695

[28] Guengerich FP. Mechanisms of Cytochrome P450-Catalyzed Oxidations. ACS Catal. 2018;8(12):10964–76. 31105987 10.1021/acscatal.8b03401 PMC6519473

[29] Nguyen T-D, Dang T-TT. Cytochrome P450 Enzymes as Key Drivers of Alkaloid Chemical Diversification in Plants. Front Plant Sci. 2021;12:12682181–682181. 10.3389/fpls.2021.682181 PMC8336426 34367208

[30] Besseau S, Kellner F, Lanoue A, Thamm AM, Salim V, Schneider B, Geu-Flores F, Höfer R, Guirimand G, Guihur A, et al. A pair of tabersonine 16-hydroxylases initiates the synthesis of vindoline in an organ-dependent manner in Catharanthus roseus. Plant Physiol. 2013;163(4):1792–803. 24108213 10.1104/pp.113.222828 PMC3850188

[31] Irmler S, Schröder G, St-Pierre B, Crouch NP, Hotze M, Schmidt J, Strack D, Matern U, Schröder J. Indole alkaloid biosynthesis in Catharanthus roseus: new enzyme activities and identification of cytochrome P450 CYP72A1 as secologanin synthase. Plant J. 2000;24(6):797–804. 11135113 10.1046/j.1365-313x.2000.00922.x

[32] Yang Y, Li W, Pang J, Jiang L, Qu X, Pu X, Zhang G, Luo Y. Bifunctional Cytochrome P450 Enzymes Involved in Camptothecin Biosynthesis. ACS Chem Biol. 2019;14(6):1091–6. 31117393 10.1021/acschembio.8b01124

[33] Kilgore MB, Augustin MM, May GD, Crow JA, Kutchan TM. CYP96T1 of Narcissus sp. aff. pseudonarcissus Catalyzes Formation of the Para-Para’ C-C Phenol Couple in the Amaryllidaceae Alkaloids. Front Plant Sci 2016;7225. 10.3389/fpls.2016.00225 PMC4766306 26941773

[34] Du Z, Peng Z, Yang H, Wu H, Sun J, Huang L. Identification and functional characterization of three cytochrome P450 genes for the abietane diterpenoid biosynthesis in Isodon lophanthoides. Planta. 2023;257(5):90. 36991182 10.1007/s00425-023-04125-z

[35] Jia H, Tan S, Cai Y, Guo Y, Shen J, Zhang Y, Ma H, Zhang Q, Chen J, Qiao G, et al. Low-input PacBio sequencing generates high-quality individual fly genomes and characterizes mutational processes. Nat Commun. 2024;15(1):5644. 38969648 10.1038/s41467-024-49992-6 PMC11226609

[36] Athanasopoulou K, Boti MA, Adamopoulos PG, Skourou PC, Scorilas A. Third-Generation Sequencing: The Spearhead towards the Radical Transformation of Modern Genomics. Life (Basel) 2021;12(1):30. 10.3390/life12010030 PMC8780579 35054423

[37] Zhu G, Wang S, Huang Z, Zhang S, Liao Q, Zhang C, Lin T, Qin M, Peng M, Yang C, et al. Rewiring of the Fruit Metabolome in Tomato Breeding. Cell. 2018;172(1):249–e261212. 29328914 10.1016/j.cell.2017.12.019

[38] Luo H, Liu H, Zhang J, Hu B, Zhou C, Xiang M, Yang Y, Zhou M, Jing T, Li Z, et al. Full-length transcript sequencing accelerates the transcriptome research of Gymnocypris namensis, an iconic fish of the Tibetan Plateau. Sci Rep. 2020;10(1):9668. 32541658 10.1038/s41598-020-66582-w PMC7296019

[39] Langmead B, Salzberg SL. Fast gapped-read alignment with Bowtie 2. Nat Methods. 2012;9(4):357–9. 22388286 10.1038/nmeth.1923 PMC3322381

[40] Li WZ, Jaroszewski L, Godzik A. Tolerating some redundancy significantly speeds up clustering of large protein databases. Bioinformatics. 2002;18(1):77–82. 11836214 10.1093/bioinformatics/18.1.77

[41] Tatusov RL, Fedorova ND, Jackson JD, Jacobs AR, Kiryutin B, Koonin EV, Krylov DM, Mazumder R, Mekhedov SL, Nikolskaya AN et al. The COG database: an updated version includes eukaryotes. BMC Bioinformatics 2003;4:41. 10.1186/1471-2105-4-41 PMC222959 12969510

[42] Bairoch A, Apweiler R. The SWISS-PROT protein sequence database and its supplement TrEMBL in 2000. Nucleic Acids Res. 2000;28(1):45–8. 10592178 10.1093/nar/28.1.45 PMC102476

[43] Kanehisa M, Goto S, Kawashima S, Okuno Y, Hattori M. The KEGG resource for deciphering the genome. Nucleic Acids Res. 2004;32:D277–80. 14681412 10.1093/nar/gkh063 PMC308797

[44] Ashburner M, Ball CA, Blake JA, Botstein D, Butler H, Cherry JM, Davis AP, Dolinski K, Dwight SS, Eppig JT, et al. Gene Ontology: tool for the unification of biology. Nat Genet. 2000;25(1):25–9. 10802651 10.1038/75556 PMC3037419

[45] Shimizu K, Adachi J, Muraoka Y. ANGLE: a sequencing errors resistant program for predicting protein coding regions in unfinished cDNA. J Bioinform Comput Biol. 2006;4(3):649–64. 16960968 10.1142/s0219720006002260

[46] Love MI, Huber W, Anders S. Moderated estimation of fold change and dispersion for RNA-seq data with DESeq2. Genome Biol 2014, 15(12). 10.1186/s13059-014-0550-8 PMC4302049 25516281

[47] Wretensjö I, Karlberg B. Characterization of sterols in refined borage oil by GC-MS. J Am Oil Chem Soc. 2002;79(11):1069–74.

[48] Zhang X, Cambrai A, Miesch M, Roussi S, Raul F, Aoude-Werner D, Marchioni E. Separation of Delta5- and Delta7-phytosterols by adsorption chromatography and semipreparative reversed phase high-performance liquid chromatography for quantitative analysis of phytosterols in foods. J Agric Food Chem. 2006;54(4):1196–202. 16478236 10.1021/jf052761x

[49] Yang B, Karlsson RM, Oksman PH, Kallio HP. Phytosterols in sea buckthorn (Hippophae rhamnoides L.) berries: identification and effects of different origins and harvesting times. J Agric Food Chem. 2001;49(11):5620–9. 11714369 10.1021/jf010813m

[50] Kumar P, Kumar D, Pal S, Singh S. Plant secondary metabolites in defense against phytopathogens: Mechanisms, biosynthesis, and applications. Physiol Mol Plant Pathol. 2025;138:102639.

[51] Xiang M-L, Hu B-Y, Qi Z-H, Wang X-N, Xie T-Z, Wang Z-J, Ma D-Y, Zeng Q, Luo X-D. Chemistry and bioactivities of natural steroidal alkaloids. Nat Prod Bioprospecting. 2022;12(1):23. 10.1007/s13659-022-00345-0 PMC9198197 35701630

[52] Lipko A, Pączkowski C, Perez-Fons L, Fraser PD, Kania M, Hoffman-Sommer M, Danikiewicz W, Rohmer M, Poznanski J, Swiezewska E. Divergent contribution of the MVA and MEP pathways to the formation of polyprenols and dolichols in Arabidopsis. Biochem J. 2023;480(8):495–520. 37022297 10.1042/BCJ20220578 PMC10212524

[53] Pu X, Dong X, Li Q, Chen Z, Liu L. An update on the function and regulation of methylerythritol phosphate and mevalonate pathways and their evolutionary dynamics. J Integr Plant Biol. 2021;63(7):1211–26. 33538411 10.1111/jipb.13076

[54] Kellner F, Geu-Flores F, Sherden NH, Brown S, Foureau E, Courdavault V, O’Connor SE. Discovery of a P450-catalyzed step in vindoline biosynthesis: a link between the aspidosperma and eburnamine alkaloids. Chem Commun. 2015;51(36):7626–8. 10.1039/c5cc01309g 25850027

[55] Nett RS, Lau W, Sattely ES. Discovery and engineering of colchicine alkaloid biosynthesis. Nature. 2020;584(7819):148–53. 32699417 10.1038/s41586-020-2546-8 PMC7958869

[56] Jiang Y, Peng W, Li Z, You C, Zhao Y, Tang D, Wang B, Li S. Unexpected Reactions of α,β-Unsaturated Fatty Acids Provide Insight into the Mechanisms of CYP152 Peroxygenases. Angew Chem Int Ed. 2021;60(46):24694–701. 10.1002/anie.202111163 34523786

[57] Fujiyama K, Hino T, Kanadani M, Watanabe B, Jae Lee H, Mizutani M, Nagano S. Structural insights into a key step of brassinosteroid biosynthesis and its inhibition. Nat Plants. 2019;5(6):589–94. 31182839 10.1038/s41477-019-0436-6

[58] Fujita S, Ohnishi T, Watanabe B, Yokota T, Takatsuto S, Fujioka S, Yoshida S, Sakata K, Mizutani M. Arabidopsis CYP90B1 catalyses the early C-22 hydroxylation of C27, C28 and C29 sterols. Plant J. 2006;45(5):765–74. 16460510 10.1111/j.1365-313X.2005.02639.x

[59] Burris-Hiday SD, Scott EE. Steroidogenic cytochrome P450 17A1 structure and function. Mol Cell Endocrinol. 2021;528:111261. 33781841 10.1016/j.mce.2021.111261 PMC8087655

