# Supplementary material for: Integrated metabolomics and transcriptomics investigate the bulb-specific biosynthesis of medicinal steroidal alkaloids in Fritillaria taipaiensis
Source: BMC Plant Biol. 2026 Feb 28;26:618. doi: 10.1186/s12870-026-08432-x (PMC13059530; doi:10.1186/s12870-026-08432-x)
Supplement: Supplementary file 4 — Supplementary Material 4: Table S1. Primer information. [file 12870_2026_8432_MOESM4_ESM.docx]

**Table S1. List of primers used in this study.**

| Used for gene cloning | |
| --- | --- |
| Frt1771AACT1-QRT-F | GCGGTACCATGAAGACTTTTTCAAGCTTCTTTC |
| Frt1771AACT1-QRT-R | GTCAGCATTTGATAGTGCCTTCGGT |
| Frt2164HMGR1-1-QRT-F | CCAAGATCCCGCCCAGAACG |
| Frt2164HMGR1-1-QRT-R | GGCATGGTCACGGAGACATGAA |
| Frt1582MK-QRT-F | GGAGCTGGTGGAGGAGGATGT |
| Frt1582MK-QRT-R | GACCGTTCCCACCAACTACAGC |
| Frt1707MVD-QRT-F | GCAGTCTGCTTGGACACTAGACC |
| Frt1707MVD-QRT-R | ACAGCATTTGGACCGGCATCA |
| Frt2624DXS-QRT-F | GGCGCAGCATGGGTTGAATC |
| Frt2624DXS-QRT-R | GCCGTCAAGGAGGCCATCAA |
| Frt1124ISPD-QRT-F | GGGCAAGCATGCCAAAGCAA |
| Frt1124ISPD-QRT-R | TCCTTCACTTCCGACATTCGGGATA |
| Frt1604ISPE-QRT-F | TCGACAAATGTGCCCGGTGT |
| Frt1604ISPE-QRT-R | TGATTAGCAGCCCACAAAGCAGT |
| Frt2637ISPG-QRT-F | GGCGCACCCTATTCGACCTT |
| Frt2637ISPG-QRT-R | TGCATCGGCCATCTCACCTG |
| Frt995ISPF-QRT-F | TCATCGGCGGCATCGACATC |
| Frt995ISPF-QRT-R | CCCAAGATCGCATCCACCACA |
| Frt1615SQS1-QRT-F | TGGCCTCGCGCTATATGGAGT |
| Frt1615SQS1-QRT-R | AGGCAGTCCTGAGCATGCAAC |
| Frt1927SQE1-QRT-F | CAACCCTCGCCCGATGAGTC |
| Frt1927SQE1-QRT-R | GCGAAGGAAACGGCAACAGC |
| Frt1910CYP51-QRT-F | CGCTGCATCAAGGAGGCTCT |
| Frt1910CYP51-QRT-R | CGCAACAATGTGGCCCTTCG |
| Frt1408C14R-QRT-F | AGTTGCTCGCTTCTGGTTACTGG |
| Frt1408C14R-QRT-R | ACCTAGCGTCGTCCCTCCTC |
| Frt1182DWF7-QRT-F | CCTCTTCCTGGTGCCAACCC |
| Frt1182DWF7-QRT-R | GTGGTGGATGGTGTGGTAGCC |
