# Supplementary material for: Integrated metabolomics and transcriptomics investigate the bulb-specific biosynthesis of medicinal steroidal alkaloids in Fritillaria taipaiensis
Source: BMC Plant Biol. 2026 Feb 28;26:618. doi: 10.1186/s12870-026-08432-x (PMC13059530; doi:10.1186/s12870-026-08432-x)
Supplement: Supplementary file 7 — Supplementary Material 7: The following supplementary data are available. Fig. S1. The phenotype of F. taipaiensis; Fig. S2. Multivariate analysis of metabolomic data from bulb and leaf tissues of F. taipaiensis; Fig.S3. Correlation and differential gene expression analysis in F. taipaiensis; Fig. S4. Comparison of gene expression levels in the phenylpropanoid pathway (by FPKM values) between bulbs and leaves of the F. taipaiensis; Fig.S5. The qRT-PCR validation confirms the gene expression levels derived from the transcriptome analysis; Fig.S6. Pearson correlation plot of DEGs putatively involved in the steroidal alkaloid biosynthetic pathway and steroidal alkaloid metabolites in F.taipaiensis. Fig.S7. Comparison of gene expression levels of CYP450 gene family in F. taipaiensis (by FPKM values) between bulbs and leaves; File S1 Information Table of All Metabolites in F. taipaiensis by UPLC-MS-MS; File S2 Differential Metabolites Profiling between Bulb and Leaf Tissues of F. taipaiensis; File S3 Summary of Transcriptomic Sample Quality Metrics for F. taipaiensis; File S4 KEGG enrichment of DEGs in leaf and bulb tissues of F. taipaiensis;File S5 Table of Enzyme Genes Involved in the Steroidal Alkaloid Biosynthesis Pathway of F. taipaiensis. [file 12870_2026_8432_MOESM7_ESM.pdf]

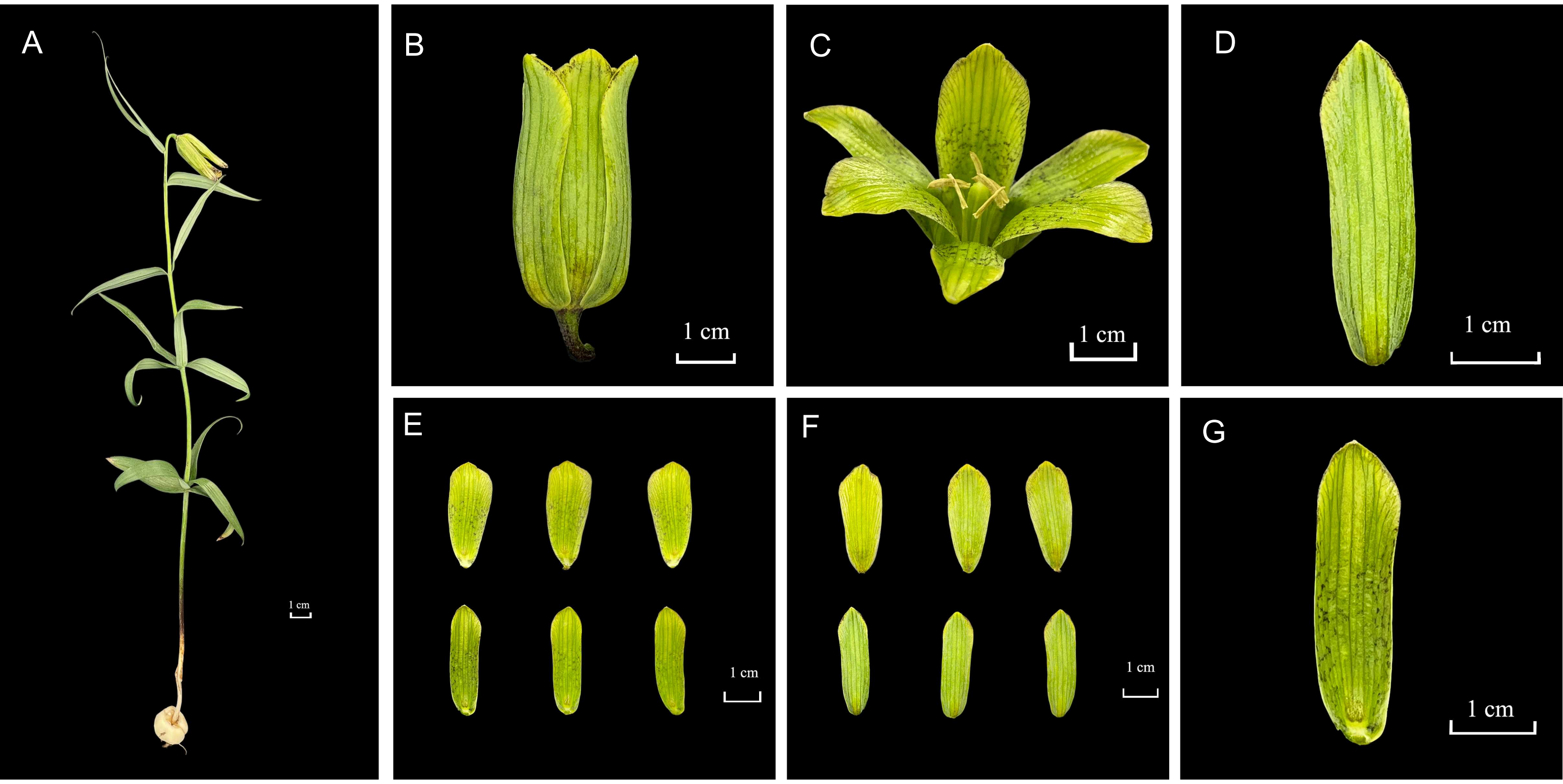

Figure S1. The phenotype of *F. taipaiensis*. A. The whole plant of *F. taipaiensis.* Scale bar,1.0cm. B. The *F. taipaiensis* Flowers. Scale bar, 1.0 cm. C. The flower of *F. taipaiensis* (The tepal is artificially peeled off to show the pistil and staments). Scale bar, 1.0cm. D. The exterior of outer perianth Scale bar, 1.0cm. (E-F) The perianth segment. E. The inner side F. The exterior side. Scale bar, 1.0cm. G. The interior of outer perianth. Scale bar, 1.0cm.

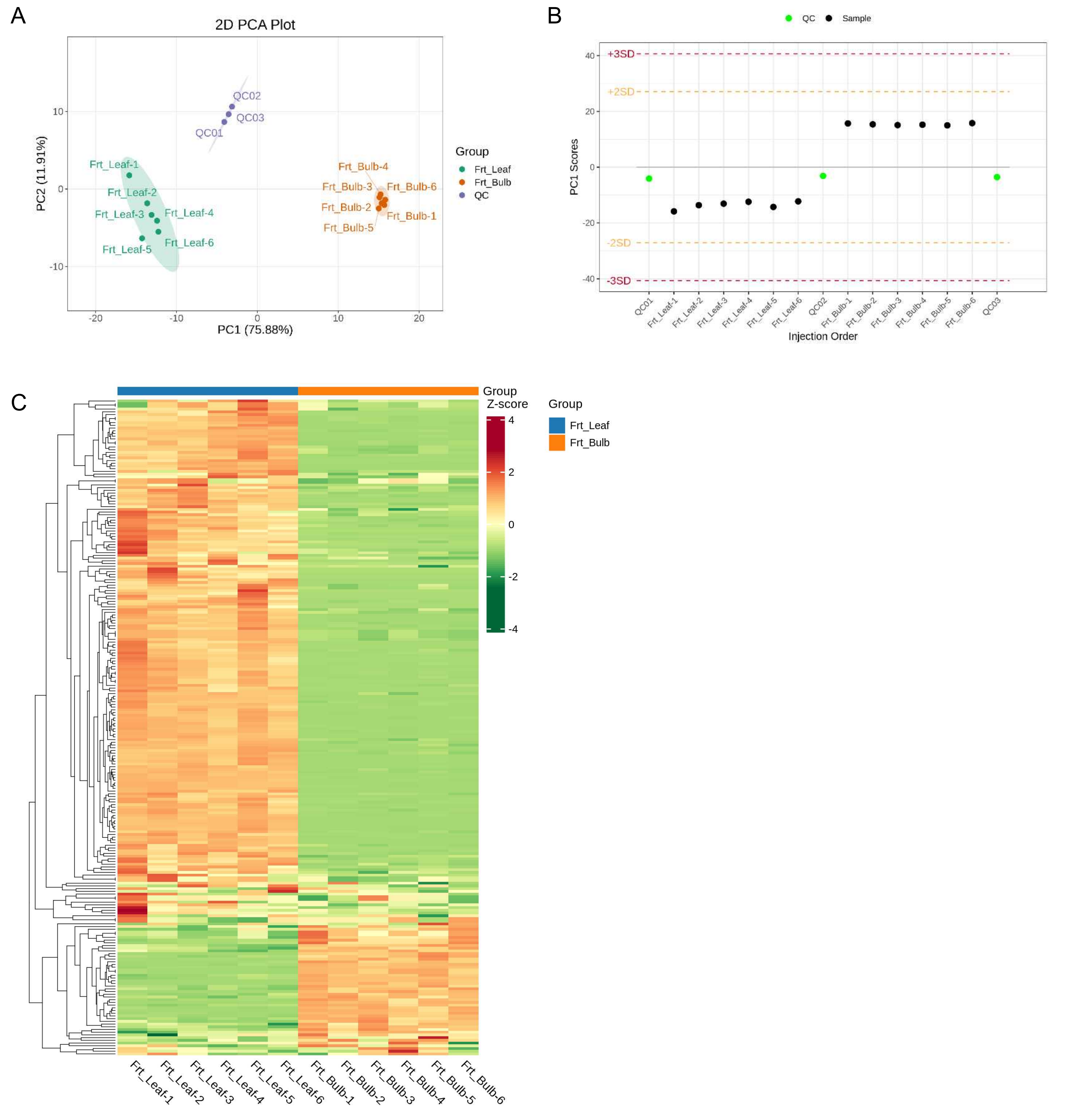

Figure S2. Multivariate analysis of metabolomic data from bulb and leaf tissues of *F. taipaiensis*. A. Principal component analysis (PCA) of metabolic profiles from bulb and leaf tissues. The horizontal axis (PC1) represents the principal component capturing the largest variance, indicating differences between groups; the vertical axis (PC2) represents the orthogonal component reflecting variance within groups. Percent values indicate the proportion of variance explained by each component. Each point corresponds to a sample, with samples colored by tissue type.B. Quality control chart based on PC1 values. The abscissa represents the sample injection sequence, and the ordinate shows the PC1 value. The yellow and red lines indicate ±2 and ±3 standard deviations, respectively. Green dots represent quality control (QC) samples; black dots represent experimental samples. C. Clustering heatmap showing the relative abundance of all detected alkaloid metabolites across samples. Rows represent metabolites, columns represent samples, and color intensity reflects normalized abundance values.

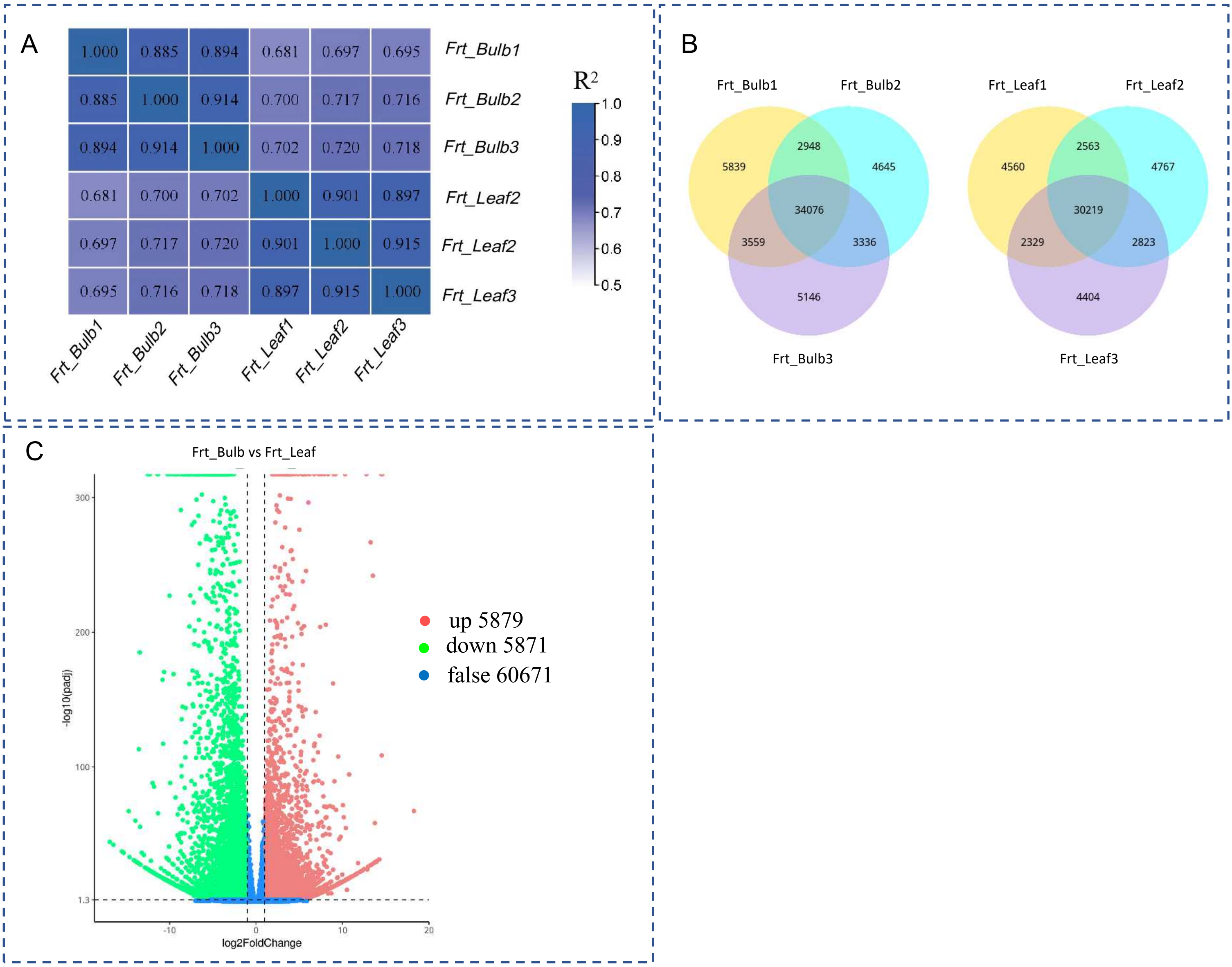

Figure S3. Correlation and differential gene expression analysis in *F. taipaiensis*. Heatmap displaying Pearson correlation coefficients among samples. TB_LJ represents bulb tissues and TB_Y represents leaf tissues of *F. taipaiensis*. B. Venn diagram showing the overlap of expressed genes between bulb and leaf samples. C. Volcano plot illustrating differentially expressed genes between tissues. Significantly upregulated and downregulated genes are highlighted based on statistical thresholds ($|log_2FC| > 1$, adjusted p-value < 0.05).

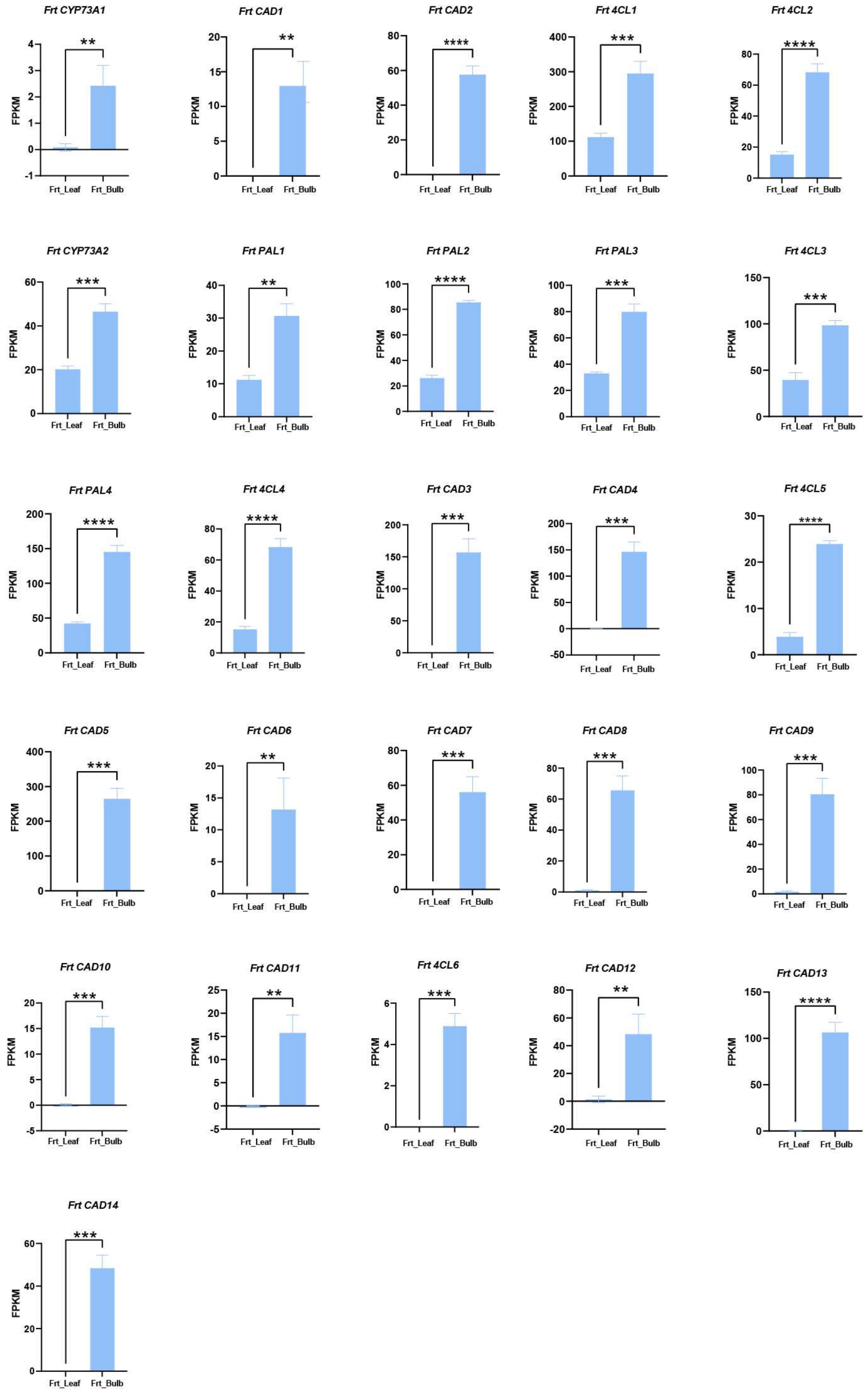

Figure S4. Comparison of gene expression levels in the phenylpropanoid pathway (by FPKM values) between bulbs and leaves of the *F. taipaiensis*. *P < 0.05, **P < 0.01, ***P < 0.001, ****P < 0.0001, ns means no significant difference (t-test).

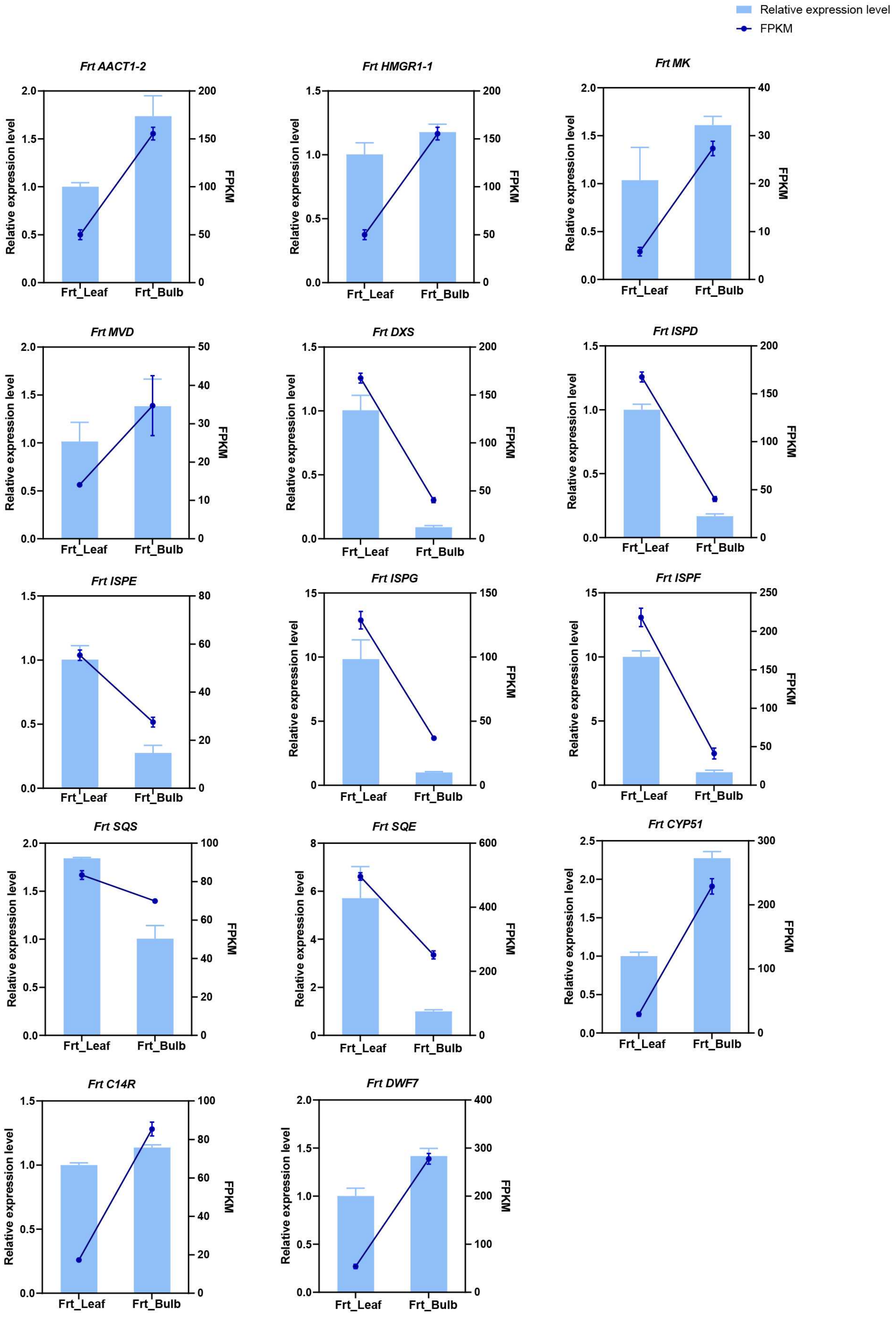

Figure S5. The qRT-PCR validation confirms the gene expression levels derived from the transcriptome analysis. The bar chart illustrates the qRT-PCR results, while the line chart represents the FPKM values. *P < 0.05, **P < 0.01, ***P < 0.001, ****P < 0.0001, ns means no significant difference (t-test).

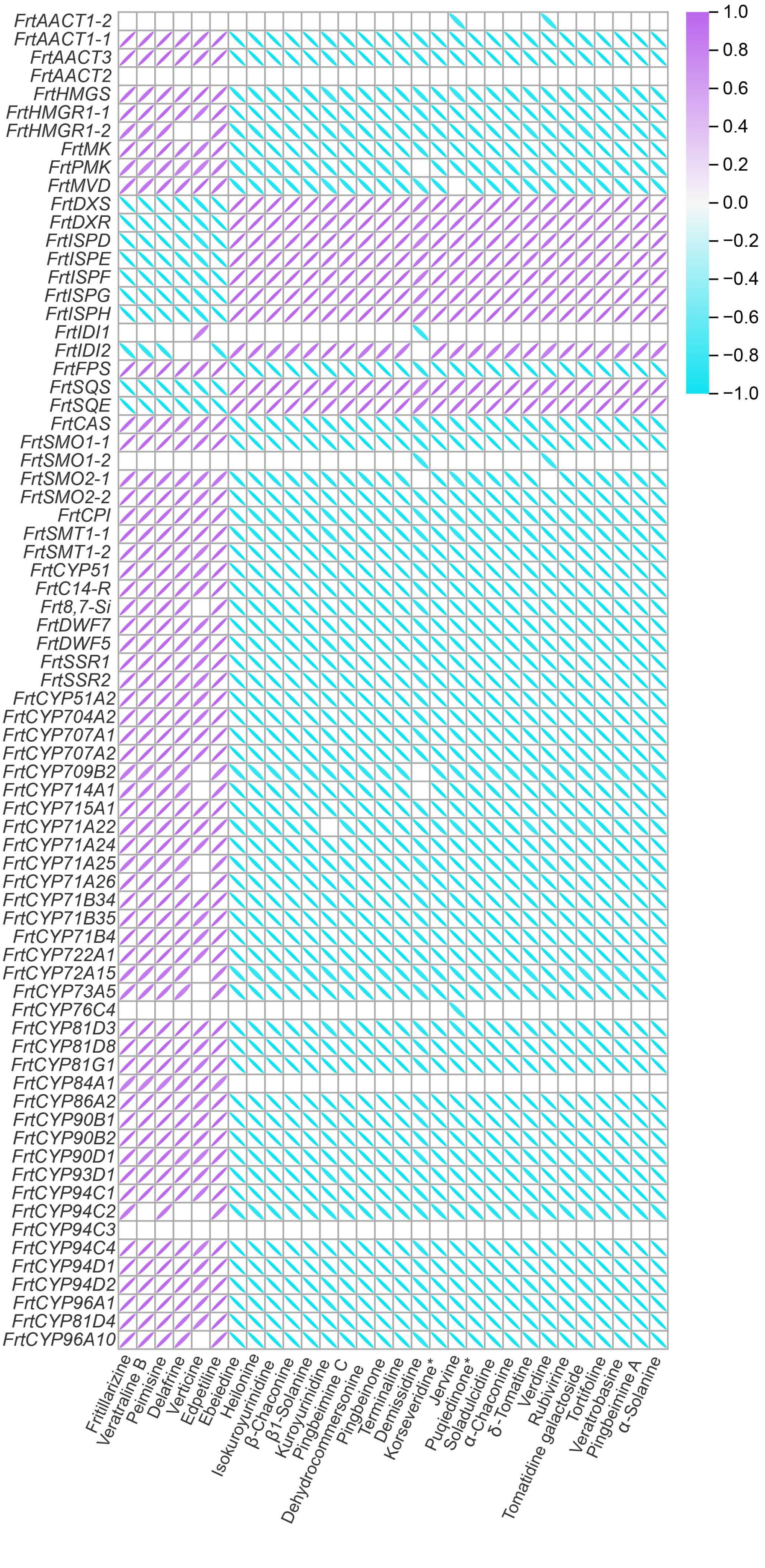

Figure S6. Pearson correlation plot of DEGs putatively involved in the steroidal alkaloid biosynthetic pathway and steroidal alkaloid metabolites in *F.taipaiensis*. Grid color represents the magnitude of correlation (purple = positive correlation, blue = negative correlation); narrower ellipses and deeper hues indicate larger absolute correlation values. White cells denote no statistically significant correlations.

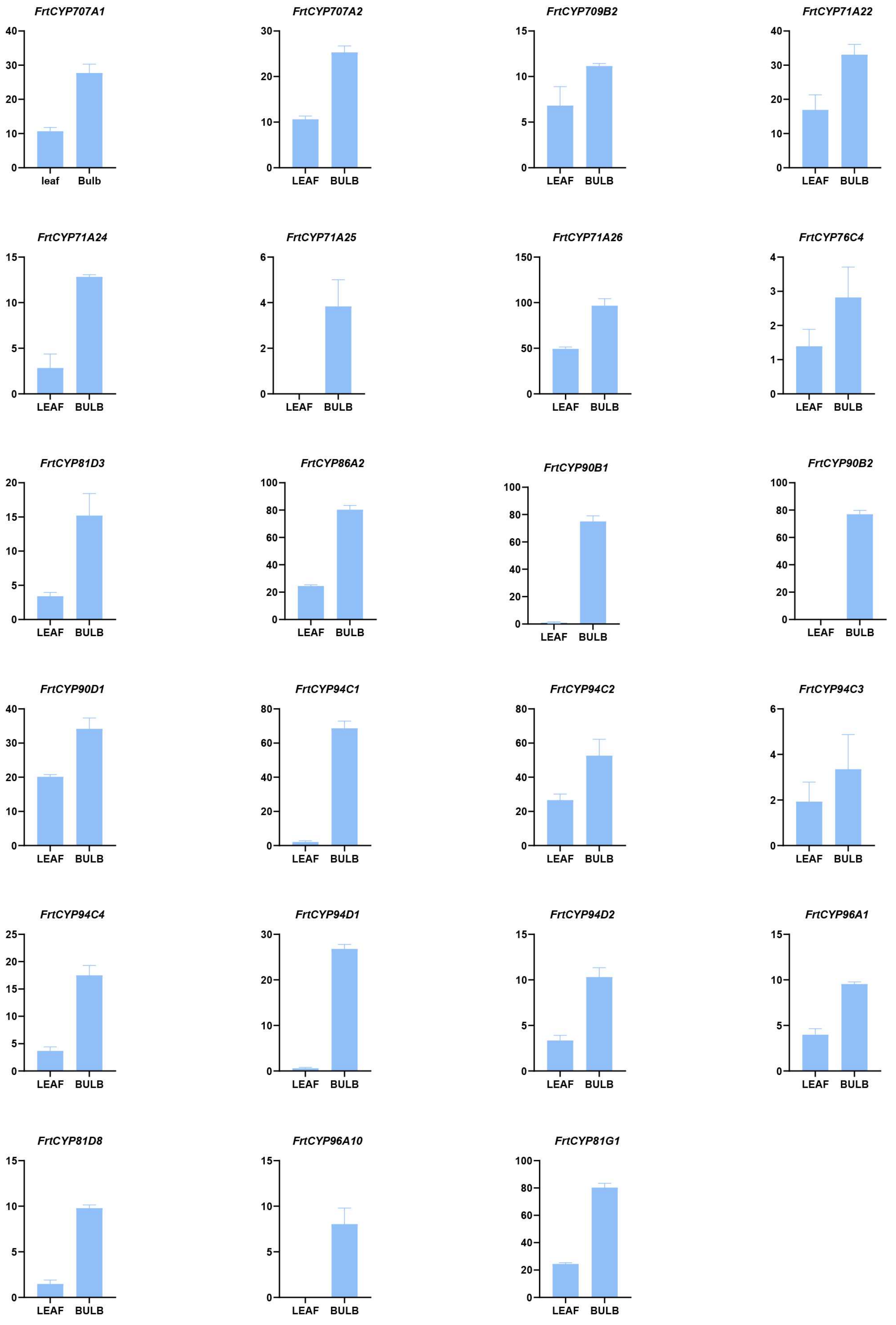

Figure S7. Comparison of gene expression levels of *CYP450* gene family in *F. taipaiensis* (by FPKM values) between bulbs and leaves. *P < 0.05, **P < 0.01, ***P < 0.001, ****P < 0.0001, ns means no significant difference (t-test).
